# Wirelessly Activated Nanotherapeutics for In Vivo Programmable Photodynamic‐Chemotherapy of Orthotopic Bladder Cancer

**DOI:** 10.1002/advs.202200731

**Published:** 2022-04-07

**Authors:** Bowen Sun, Juwita Norasmara Bte Rahmat, Han Joon Kim, Ratha Mahendran, Kesavan Esuvaranathan, Edmund Chiong, John S. Ho, Koon Gee Neoh, Yong Zhang

**Affiliations:** ^1^ Department of Chemical and Biomolecular Engineering College of Design and Engineering National University of Singapore Singapore 117585 Singapore; ^2^ Department of Biomedical Engineering College of Design and Engineering National University of Singapore Singapore 117583 Singapore; ^3^ Department of Electrical and Computer Engineering College of Design and Engineering National University of Singapore Singapore 117583 Singapore; ^4^ Department of Surgery Yong Loo Lin School of Medicine National University of Singapore Singapore 119228 Singapore; ^5^ Department of Urology National University Health System Singapore 119228 Singapore; ^6^ Institute for Health Innovation and Technology National University of Singapore Singapore 119276 Singapore; ^7^ The N.1 Institute for Health National University of Singapore Singapore 117456 Singapore

**Keywords:** bladder cancer, photodynamic therapy, targeted drug delivery, wireless photonics

## Abstract

Photochemical internalization (PCI) is a promising intervention using photodynamic therapy (PDT) to enhance the activity of chemotherapeutic drugs. However, current bladder cancer treatments involve high‐dose chemotherapy and high‐irradiance PDT which cause debilitating side effects. Moreover, low penetration of light and drugs in target tissues and cumbersome light delivery procedures hinder the clinical utility of PDT and chemotherapy combination for PCI. To circumvent these challenges, a photodynamic‐chemotherapy approach is developed comprising tumor‐targeting glycosylated nanocarriers, coloaded with chlorin e6 (Ce6) and gemcitabine elaidate (GemE), and a miniaturized implantable wirelessly powered light‐emitting diode (LED) as a light source. The device successfully delivers four weekly light doses to the bladder while the nanocarrier promoted the specific accumulation of drugs in tumors. This approach facilitates the combination of low‐irradiance PDT (1 mW cm^−2^) and low‐dose chemotherapy (≈1500× lower than clinical dose) which significantly cures and controls orthotopic disease burden (90% treated vs control, 35%) in mice, demonstrating a potential new bladder cancer treatment option.

## Graphical abstract

Nanocarriers and implanted wireless light generating device enhances therapy by photochemical internalization (PCI). Combined with low‐dose gemcitabine elaidate (GemE), photodynamic therapy (PDT) results in significant treatment response in orthotopic bladder tumors in mice.

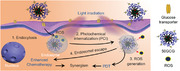

## 1 Introduction

High‐risk nonmuscle invasive bladder cancer (NMIBC) is managed by tumor resection combined with adjuvant bacillus Calmette‐Guérin (BCG) immunotherapy. However, BCG fails to control recurrences in 33% to 42% of the cases, with a 10% risk of progression to muscle‐invasive disease,^[^ ^1^ ^]^ which usually necessitates cystectomy, a procedure with well‐documented morbidities.^[^ ^2^ ^]^ Alternative therapies employ chemotherapeutic agents for intrabladder treatment, but their applications are limited due to low effectiveness.^[^ ^3^ ^]^ For example, gemcitabine and mitomycin C are two widely‐used intravesical agents, but their complete response rates are low at 21% (2 years) and 19% (3 years), respectively.^[^ ^4^ ^]^ Therefore, disease recurrence remains a dilemma for both patients and physicians. Furthermore, high concentrations of intravesical agents are associated with side effects such as irritative bladder symptoms, hematuria, and painful voiding.^[^ ^5^ ^]^ Hence, there is an urgent demand for treatments that can effectively eradicate bladder tumors while minimizing the side effects.

Photodynamic therapy (PDT) was once considered a potentially promising treatment that yielded a high response between 75% and 84% at first surveillance and a complete response of 53%.^[^ ^6^ ^]^ However, PDT failed to gain traction due to the high percentage of patients exhibiting adverse events (AEs) such as incontinence, irritative symptoms, and permanent bladder contracture.^[^ ^6^, ^7^ ^]^ The principal reason for these AEs is the unwanted activation of the photosensitizer (PS) in normal bladder urothelium, due to their nonspecific accumulation in healthy cells.^[^ ^8^ ^]^ Moreover, intravesically administrated PS has limited retention time in the bladder, reducing their bioavailability and accumulation in tumor tissues, resulting in sub‐optimal PS activation. Current light delivery approaches using laser systems also have apparent drawbacks, mainly being bulky, costly, and may not provide the optimum wavelength for PS activation. To deliver light to deeper tumors, optical fibers are inserted via surgery or endoscopy. However, the fibers are non‐implantable and allow only single light delivery per insertion.^[^ ^9^ ^]^ Furthermore, the procedures may cause postsurgical pain or discomfort, and patients' movements are restricted during therapy.

Here, we propose a strategy for wirelessly powered in vivo photodynamic‐chemotherapy with low‐irradiance and low‐dose chemotherapy to overcome the current limitations of PDT and chemotherapy. A miniaturized implantable wireless LED device for programmable delivery of multiple doses of light over time and glycosylated nanocarriers coloaded with PDT and chemotherapy agents (Ce6 and GemE) for targeted drug and PS delivery were developed and their effectiveness was demonstrated using an orthotopic model of bladder cancer. The LED device is miniaturized (0.162 ± 0.019 g, 8 mm diameter, 2 mm thickness) for long term implantation and designed to produce optimum multiwavelength light emissions for PS activation. The wireless approach can significantly improve patients' comfort as multiple endoscopies or surgeries for fiber optics insertion are eliminated. Tumor targeting can increase the impact of PDT by protecting the normal bladder urothelium from nonspecific PS uptake and PDT damage. Hence, a glycosylated nanocarrier was used to target glucose transporters (GLUTs), which are overexpressed by cancer cells due to the Warburg effect.^[^ ^10^ ^]^ The photodynamic‐chemotherapy of Ce6 and GemE can be expected to result in treatment synergy because of the photochemical internalization (PCI) effect.^[^ ^11^ ^]^ After endocytosis, the Ce6 loaded nanocarriers generate reactive oxygen species (ROS) and break the endosomes under low dose light irradiation, allowing coloaded GemE to readily cross the intracellular barriers and exert its chemotherapeutic effect more effectively.

Our wirelessly powered LED device activated the PS delivered intravesically in targeting nanocarriers coloaded with a chemotherapy drug over four instillations in an orthotopic murine model of bladder cancer. This approach offered significant treatment response rates compared to the control (35% vs 90% for wirelessly powered photodynamic‐chemotherapy. Our strategy could improve treatment outcomes and potentially increase patients' post‐therapy comfort and satisfaction by reducing debilitating AEs associated with high dose drugs and high irradiance PDT.

## 2 Results

### 2.1 Nanocarrier Synthesis and Characterization

**Figure 1:**
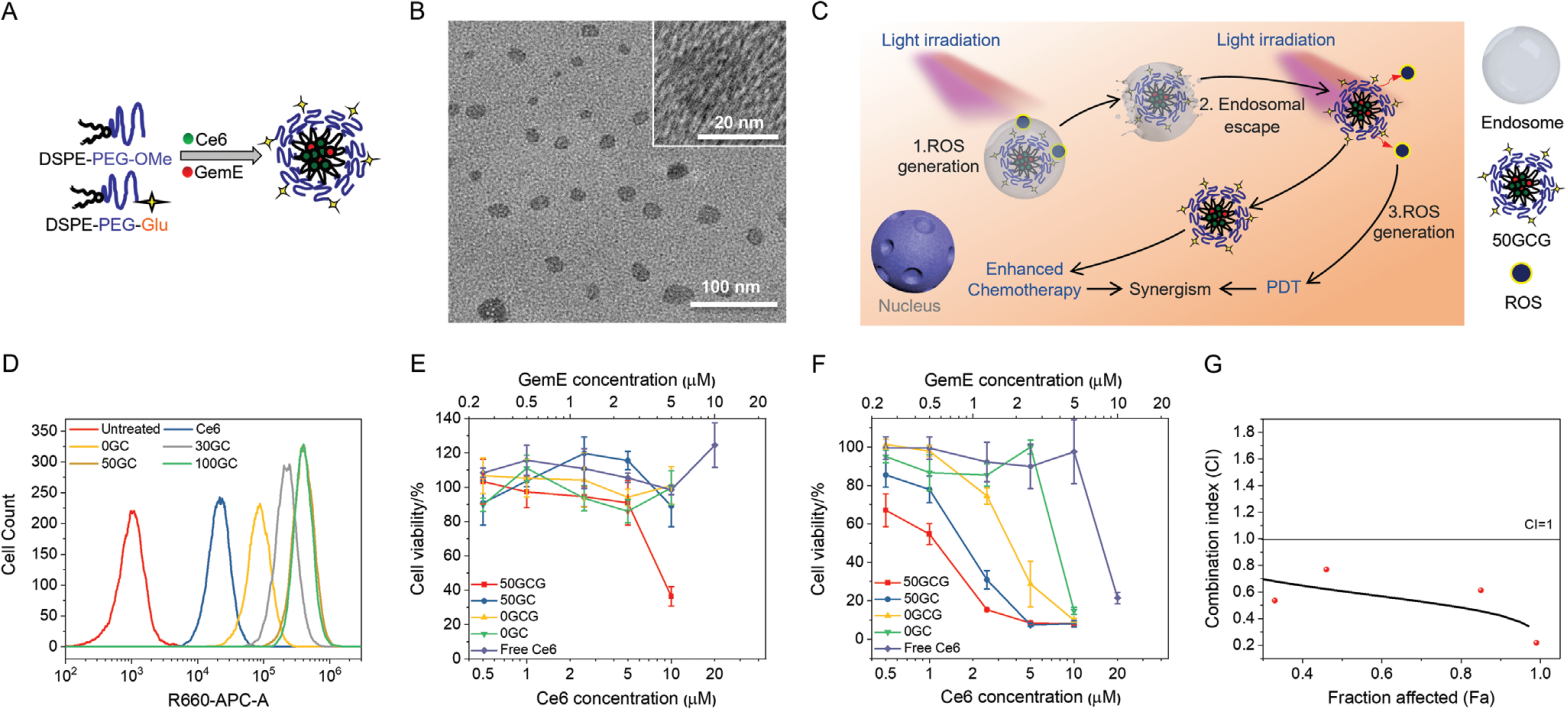
Fabrication of Ce6 and GemE coloaded glycosylated nanocarriers and their in vitro targeting and therapeutic effects for bladder cancer treatment. A) Schematic illustration of the fabrication process of Ce6 and GemE coloaded glycosylated nanocarriers. B) TEM and HRTEM images of Ce6 and GemE coloaded glycosylated nanocarrier, 50GCG. C) Schematic illustration of the mechanism of Ce6 and GemE coloaded glycosylated carrier for a synergistic combination of PDT and chemotherapy because of PCI effect. D) Flow cytometry analysis of cellular uptake of Ce6‐loaded DSPE‐PEG glycosylated carriers with different percentages of DSPE‐PEG‐Glu (as indicated by the number in the sample name) after 2 h incubation. UMUC3 viability after 2 h incubation with 50GCG, 50GC, 0GCG, 0GC, and free Ce6 E) without and F) with 660 nm laser irradiation (250 mW cm^−2^) for 5 min. Data are presented as mean ± SD (*n* = 3). G) CI versus Fa plot of 50GCG with 660 nm laser irradiation against UMUC3 cells. CI values were calculated using the cell viability results obtained with 50GCG with and without light irradiation and 50GC with light irradiation.

DSPE‐PEG‐Glu was synthesized by conjugating the –NH_2_ group of glucosamine to the –COOH group of DSPE‐PEG‐COOH using EDC/NHS (Figure S1, Supporting Information) The glycosylated DSPE‐PEG micellar carriers were formed using the cosolvent method.^[^ ^12^ ^]^ The hydrophobic Ce6 and GemE were coloaded in the hydrophobic DSPE core of the micelle (**Figure** **1**A). The transmission electron microscopy (TEM) and high‐resolution transmission electron microscopy (HRTEM) images of Ce6 and GemE coloaded DSPE‐PEG carriers (Figure 1B (glycosylated nanocarrier) and Supporting Information, Figure S2A in the Supporting Information (nonglycosylated nanocarrier) indicated that the micelles are spherical with sizes between ≈10 and 20 nm, which is considered optimal for cellular uptake and avoiding extravasation.^[^ ^13^ ^]^ These carriers can be stably stored at 4 °C for an extended period. The TEM images of the Ce6 and GemE coloaded carriers (50GCG and 0GCG) stored at 4 °C for 258 d showed that after this period, these carriers still maintained their micellar structure and particle size (Figure S2B,C, Supporting Information). In addition, these carriers (glycosylated and nonglycosylated) have a negative surface charge (Figure S2D, Supporting Information), which will reduce interactions and random adhesions to the negatively charged healthy urothelium. The encapsulation efficacies (EE) of coloaded Ce6 and GemE are both >80%. The loading efficiencies (LE) of Ce6 singly loaded in the carriers (0GC and 50GC) are similar to when it is coloaded with GemE (0GCG and 50GCG, **Table** **1**). The loading of Ce6 in the different carriers resulted in some degree of self‐quenching as indicated by the decrease in fluorescence compared to free Ce6 of the same concentration (Figure S2E, Supporting Information).

**Table 1:** LE and EE of loaded agents (Ce6, GemE) in 50GCG (glycosylated carrier with Ce6 and GemE), 50GC (glycosylated carrier with Ce6), 0GCG (nonglycosylated carrier with Ce6 and GemE), and 0GC (nonglycosylated carrier with Ce6) and their IC_50_ values against UMUC3 and MB49 cells after 660 nm laser irradiation. The IC_50_ value of free Ce6 is given for comparison

Sample name	50GCG	50GC	0GCG	0GC	Free Ce6
EE (Ce6) [%]	82.5	81.3	83.8	83.9	NA^a)^
LE (Ce6) [%]	5.0	4.9	5.0	5.0	NA
EE (GemE) [%]	91.2	NA	88.6	NA	NA
LE (GemE) [%]	2.7	NA	2.7	NA	NA
IC_50_ (Ce6)^b)^ [× 10^−6^ M] (UMUC3)	1.1	1.9	3.6	7.6	15.6
IC_50_ (GemE)^c)^ [× 10^−6^ m] (UMUC3)	0.55	NA	1.8	NA	NA
IC_50_ (Ce6) [× 10^−6^ m] (MB49)	0.50	2.23	0.82	4.11	1.71
IC_50_ (GemE) [× 10^−6^ m] (MB49)	0.25	NA	1.1	NA	NA

^a)^ NA: not applicable;

^b)^ IC_50_ (Ce6): Concentration of Ce6 required to kill 50% of the cells;

^c)^ IC_50_ (GemE): Concentration of GemE required to kill 50% of the cells.

The Ce6 release profiles from 50GC and 50GCG are similar and only about 17% of the loaded Ce6 was released in the first 2 h, which is the expected dwell time for drugs in the bladder in intravesical treatment for NMIBC (Figure S2F, Supporting Information). The release of GemE from the carriers was even slower, and the concentration of released GemE after 48 h incubation in artificial urine was lower than the detection limit of the HPLC. The slow release of GemE from the carriers is due to the strong interaction of its fatty acid chain with the aliphatic chains of DSPE. Thus, most of the loaded Ce6 and GemE are expected to remain encapsulated in the carriers before cellular uptake when used in bladder cancer therapy.

### 2.2 In Vitro Targeting

We hypothesized that the targeted photodynamic‐chemotherapy approach will result in treatment synergy due to the PCI effect. The Ce6 and GemE‐loaded glycosylated micelles interact with the overexpressed GLUTs on the bladder cancer cell membrane, promoting their selective internalization and accumulation. Activation of the Ce6 under light irradiation generates ROS, breaking the endosomes,^[^ ^11^, ^14^ ^]^ allowing the drug‐loaded micelles to escape and cross the intracellular barriers.^[^ ^14^ ^]^ ROS generated under continued light irradiation will induce PDT, while the GemE‐loaded micelles will be metabolized in the cytoplasm, thereby enhancing the chemotherapy effect (Figure 1C).

To establish the optimal glycosylation level, the in vitro targeting efficacies of the micelles with different DSPE‐PEG‐Glu content was investigated using UMUC3 bladder cancer cell line. Cells treated with nonglycosylated carriers loaded with Ce6 (0GC) exhibited increased intracellular Ce6 compared to free Ce6 treated samples. Increasing the DSPE‐PEG‐Glu content from 20% to 50% led to a concomitant increase in Ce6 uptake (Figure 1D). Ce6 uptake levels plateau with DSPE‐PEG‐Glu content beyond 50% (Figure 1D; Figure S3, Supporting Information). Therefore, DSPE‐PEG‐Glu content of 50% (50GC or 50GCG) was selected as targeting formulation for further investigations. Confocal laser scanning microscopy (CLSM) images of UMUC3 cells incubated with 0GCG, 50GCG, or free Ce6 for 2 h corroborated with the flow cytometry data (Figure S4, Supporting Information). Similar tests carried out with MB49 murine bladder cancer cell line, which is used for our orthotopic mouse model, produced comparable results to UMUC3 (Figure S5, Supporting Information).

### 2.3 In Vivo Targeting and In Vitro Therapy

The in vivo targeting efficacy of the carriers was investigated with orthotopically implanted carboxyfluorescein succinimidyl ester (CFSE)‐labeled MB49‐PSA (prostate‐specific antigen) cells. Mice intrabladderly treated with 0GC for 2 h displayed nonspecific Ce6 fluorescence in healthy tissues and minimal colocalization of the Ce6 and CFSE fluorescence from tumor cells (Pearson correlation *p* value = 0.04, Figure S6, Supporting Information). In contrast, treatment with 50GC resulted in higher colocalization of Ce6 and CFSE fluorescence (*p*‐value = 0.35) with a low Ce6 distribution in healthy tissues.

We compared the in vitro therapeutic efficacies of 0GC, 0GCG, 50GC, 50GCG, and free Ce6 for 2 h using UMUC3 cells. Under dark conditions, carriers loaded with Ce6 only (0GC, 50GC) and free Ce6 did not induce significant toxicity (Figure 1E), showing that the DSPE‐PEG carriers and Ce6 in the absence of light irradiation are not cytotoxic.^[^ ^15^ ^]^ In contrast, significant toxicity was achieved after 660 nm laser irradiation for 5 min with all formulations in a dose‐dependent manner (Figure 1F). Dark toxicity was observed in 50GCG treated cells at (GemE) concentrations higher than 2.5 × 10^−6^ M, which is attributed to the chemotherapeutic effect of GemE alone. In comparison, minimal dark toxicity was observed in 0GCG treated cells at 2.5 × 10^−6^ M (GemE) and above due to the lower uptake of 0GCG. The killing efficacies of Ce6‐loaded or Ce6 and GemE coloaded glycosylated carriers after laser irradiation are higher than the corresponding nonglycosylated carriers as shown by the IC_50_ values (Ce6) in Table 1 (50GCG (1.1 × 10^−6^ M) versus 0GCG (3.6 × 10^−6^ M), and 50GC (1.9 × 10^−6^ M) versus 0GC (7.6 × 10^−6^ M)). The higher killing efficacies in the loaded glycolsylated micelles could be attributed to higher intracellular ROS production (Figure S7, Supporting Information) due to increased uptake of the glycosylated carriers. The in vitro light toxicity results corroborated with the live/dead staining assay (Figure S8, Supporting Information). Additionally, comparing the IC_50_ of 50GCG versus 50GC or 0GCG versus 0GC showed a two‐fold IC_50_ decrease, demonstrating the enhanced effect of the photodynamic‐chemotherapy approach compared to PDT alone. In vitro cell viability experiments conducted with MB49 cells showed similar results as those obtained with UMUC3 cells (Table 1). The combination effect (synergistic, antagonist, or additive) of the targeted photodynamic‐chemotherapy strategy was evaluated (Figure 1G) from the cell viability results of the glycosylated formulations. The calculated CI values are lower than 1 at all Fa values, indicating a synergistic effect from the combination therapy. To further understand the mechanism behind the synergistic effects of the combination therapy, CLSM was performed on the cells subjected to different treatments. Without irradiation, the fluorescence of Ce6 in the 50GCG‐treated cells overlapped predominantly with the green DND‐26‐stained endosomes (Figure S9, Supporting Information). However, after 660 nm laser irradiation, the green DND‐26‐stained endosome granules were damaged and Ce6 fluorescence was also detected in the cytoplasm (Figure S9, Supporting Information). These results indicated that the PCI effect enabled the 50GCG micelles to escape the endosomal barrier into the cytoplasm. In the cytoplasm, the GemE would be hydrolyzed to gemcitabine, which is subsequently phosphorylated and transported to the nuclei or mitochondria where it is incorporated into DNA chains, resulting in chain termination and ultimately cell death.^[^ ^16^ ^]^ Since endosomal escape facilitates gemcitabine in reaching the target sites, its chemotherapeutic effect would be enhanced.^[^ ^11^, ^17^ ^]^ Thus, ROS generated by Ce6‐mediated PDT not only has direct toxic effects on the 50GCG‐treated cells, it also indirectly promotes chemotherapy by the coloaded GemE, thereby resulting in treatment synergy. Similar treatment synergy was observed in MB49 cells treated with the loaded glycosylated micelles (Figure S10, Supporting Information). We then proceeded to test the efficacy of these formulations in an orthotopic murine bladder cancer model combined with an implantable wireless micro‐LED device as a light source.

### 2.4 Characterization of the Wireless Device System

**Figure 2:**
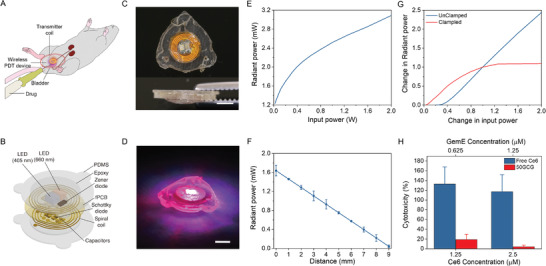
Wireless micro‐LED device for treating orthotopic bladder tumors in mice. A) Schematic of treatment strategy in mice orthotopic bladder cancer model. B) Schematic of the structure of the wireless PDT device. C) Top and side view of the implantable wireless micro‐LED device. Scale bar, 2.5 mm. D) Activated implantable device by wireless powering. Scale bar, 2.5 mm. E) Total radiant power of a single device powered by inductive coupling. F) Total radiant power delivered as a function of the distance between the device and transmitter coil. The device was powered in the inductive coupling configuration with an output power of 200 mW. Data represented as mean ± SD (*n* = 3 technical trials). G) Normalized radiant power as a function of normalized input power with and without Zener clamp to regulate light emission. H) PDT induced toxicity in MB49 cells after 2 h of 50GCG or free Ce6 treatment combined with irradiation using wireless dual micro‐LED (405/660 nm) light source at a wireless activation input power of 100 mW. Data were represented as mean ± SD. The experiment was done twice (*n* = 4).

A wireless LED device was fabricated as an in vivo light source for activating the PS encapsulated in the glycosylated micelles functioning as an intravesical delivery agent. The treatment strategy involves implanting the wireless LED device, powered using an external transmitter coil during therapy for PS activation, adjacent to the mouse bladder (**Figure** **2**A). Double coating with epoxy and polydimethylsiloxane (PDMS,a biocompatible and flexible material), protects the electronics from mechanical and chemical damages (Figure 2B). The epoxy prevents detaching or breaking of the electronics by mechanical shock, while PDMS blocks the inflow of body fluid. Three designated suture holes around the device prevent unexpected perforation damages during suturing. These packaging strategies helped improve the device survival rate during the long‐term in vivo test (33 d). The total diameter and height of the device are 8 and 2 mm, respectively (Figure 2C). The detailed fabrication procedure is described in Figure S11 in the Supporting Information.

The device emits violet and red light (Figure 2D) at wavelengths corresponding to Ce6 absorption peaks, 405 and 660 nm.^[^ ^18^ ^]^ The transmitter comprises a parallel‐connected coil array for simultaneous activation of five devices (Figure S12A, Supporting Information) and an L‐type matching network for impedance matching with a signal generator. In the wireless devices, the received power through the coil is supplied to two serially connected LEDs after rectification and boosting the voltage (Figure S12B, Supporting Information). All the implantable devices can provide irradiation of almost uniform light intensity (Figure S12C, Supporting Information). Both the transmitter coil array and receiver coil are well resonated at 50 MHz, which is a desired operating frequency (Figure S12D, Supporting Information), by selected parameters. The design parameters are summarized in Table S1 in the Supporting Information. According to a previous analysis,^[^ ^18^ ^]^ the total radiant power of the implanted device is required to exceed 1.3 mW to achieve 1 mW cm^−2^ light intensity around the device, which is sufficient to activate most PSs within 30 min.^[^ ^19^ ^]^ In the inductive coupling configuration, the wireless powering system can deliver above the required radiant power via the implanted device at 200 mW of input power (Figure 2E). Considering the implantation of the device under the subcutaneous tissues, it can produce sufficient radiant power with less than 3 mm embedded depth, as shown in Figure 2F. Controlling light dose in the target region is a challenging issue in wireless implanted devices with the variation of irradiation power depending on input power and geometry of the system. A clamping circuit that can limit supplying power to LED is a simple solution to deliver light to a target. In the presented device, the clamping circuit limited the rectified voltage to sustain 1.5 mW of the total radiant power. As a result, the light output variation is reduced from 40% to less than 10% around the clamping point depending on the input power, and it is also stable at higher input power (Figure 2G). The performance of the light delivery system was verified in vitro with free Ce6 and 50GCG treated MB49 cells. Wireless PDT with free Ce6 achieved minimal toxicity levels, but the combination of PDT and chemotherapy with 50GCG achieved significant cytotoxicity (Figure 2H).

### 2.5 Device Implantation Strategy

**Figure 3:**
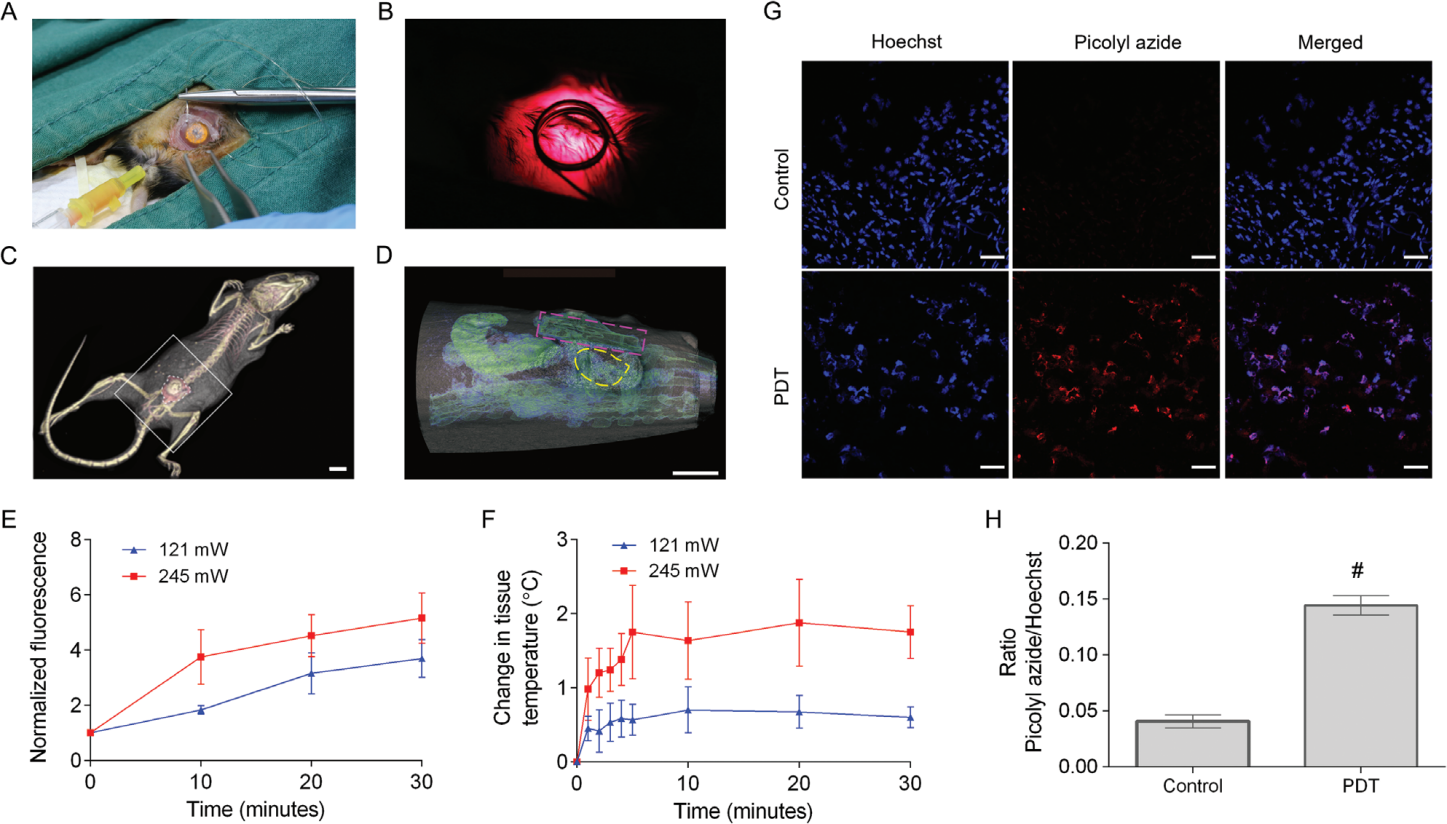
Wireless device implantation strategy. A) Photograph of the wireless device positioned on the abdominal tissue adjacent to the mouse bladder and anchored on the abdominal muscles with a few turns of the sutures. B) Photograph of the activated device after wound closure. C) CT scan of a mouse with a wireless device implanted (white dotted line) Scale bar, 10 mm. D) Magnified CT scan showing the sagittal view of the implanted device (pink dotted line) above the inflated bladder (yellow dotted line) in the lower abdominal region of the mouse (white square box in (C)) Scale bar, 5 mm. E) SOSG assay using bladder‐instilled photosensitizer/SOSG solution. F) Tissue temperature changes over time during wireless activation. The experiment was performed once (*n* = 2). Data were represented as mean ± SD. G) TUNEL assay to detect apoptosis in the bladders of tumor implanted mice after one PDT session at input power 121 mW. Blue (Hoechst 33 342) indicates stained DNA, and red (TUNEL, picolyl azide dye) indicates apoptotic cells. Objective lens, 20×. Scale bar, 50 µm. H) Mean grey areas of DNA and picolyl azide staine were measured using Image J software. The mean grey area data was represented as picolyl azide/Hoechst ratio. Data were represented as mean ± SD (*n* = 3). # *p* < 0.005.

Since the device is larger than the capacity of mice bladders, implantation in the bladder lumen is unviable. Hence, implanting the device on the abdominal muscle layer directly adjacent to the bladder under the subcutaneous layer of the skin is a suitable alternative (**Figure** **3**A). This positioning strategy is possible because mouse abdominal and bladder tissues are thin (≈0.6 mm for abdominal tissue and 1.3 mm for the whole bladder), allowing light to penetrate the inner bladder wall. The device positioning was guided by the visible full bladder (Figure S13, Supporting Information), and device activation was done before and after wound closure to confirm its operational status (Figure 3B). The procedure was minimally invasive without abdominal organ exposure, thus protecting the mice from possible infection and organ perforation. The position of the device above the pelvic area where the bladder is located was confirmed via computed tomography (CT) scans (Figure 3C,D).

**Figure 4:**
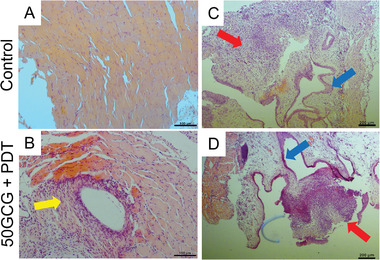
Biosafety evaluation of the implanted device and of photodynamic‐chemotherapy with glycosylated carrier. Mice were terminated after one PDT treatment (on day 18) 24 h after device activation. Hematoxylin and eosin staining of A) abdominal muscle from control mice and B) abdominal and fibrous tissue capsule surrounding the micro‐LED device in 50GCG + PDT treated mice. Objective lens, 10×. Scale bar, 100 µm. The yellow arrow indicates areas of increased nucleation in the fibrous tissue. C,D) Hematoxylin and eosin staining of bladder sections implanted with tumor. The red arrows indicate implanted tumor, and the blue arrows indicate healthy urothelium. Objective lens, 4×. Scale bar, 200 µm.

Two wireless activation settings were tested in vivo in anesthetized device‐implanted mice. For both settings, the levels of ROS produced were time‐dependent, and the rise in tissue temperature stabilized after 5 min (Figure 3E,F). The higher‐powered settings (input power 245 mW) induced greater ROS generation and higher tissue temperature increase. However, the lower‐powered settings (input power 121 mW) were sufficient to activate ROS generation within 20–30 min of continuous irradiation (normalized SOSG fluorescence levels: 3.2–3.7× compared to time 0, Figure 3E) with minimal increase in tissue temperature (maximum observed increase: 0.7 ± 0.31 °C, Figure 3F). Temperature changes of less than 1 °C do not harm healthy tissues because they are well below tissue damage thresholds set by IEEE Std. C95.1‐2005.^[^ ^20^ ^]^ The lower activation settings were used for subsequent experiments to ensure that the GemE in both groups (50GCG and 50GCG + PDT) is within the normal temperature range for fair comparisons. The efficacy of the light supply system in inducing apoptosis was evaluated in orthotopically implanted bladder tumors. After intravesical delivery of 50GCG (0.2 mg mL^−1^, 50 µL) and a 2 h drug dwelling time, PDT was performed immediately. In PDT‐treated mice, apoptotic cells were detected in the bladders 24 h after PS activation (Figure 3G). The fluorescence TUNEL staining was normalized with the nuclear Hoechst stain to account for tissue‐sectional size differences under the objective field. The normalized TUNEL stain levels were significantly elevated compared to the control, thereby demonstrating successful light delivery to the bladder for PS activation via the implantation approach. Device explantation revealed a thin (<1 mm) fibrotic capsule surrounding the device after 18 d. The fibrotic capsule looked visibly thicker at 33 d but remained at <1 mm thickness. Histological staining of the fibrotic and abdominal tissues in contact with the device did not reveal significant damage to the healthy tissues (**Figure** **4**A,B). There is a region with intense nucleation in the tissues implanted with the device (Figure 4B, yellow arrow) connected to healthy abdominal tissue layers and that is natural to the fibrotic tissues formed due to foreign body response. There were no observable device performance degradation by the foreign body response with intact PDMS encapsulation integrity, which is maintained by using only the designated suture holes during implantation procedure. As a well‐known biocompatible material and thermal insulator, PDMS, which is coated on the device, helps to minimize the foreign body effect and thermal transmission from the electronics.^[^ ^21^ ^]^ To assess the use of the device combined with PDT with 50GCG, tumor‐bearing bladder tissues after one PDT session were harvested for histological analysis. The tumor tissue in the control bladder looked intact compared to the tumor tissues in the 50GCG + PDT treated mouse (Figure 4C,D, red arrows), which appears more disintegrated after the treatment. However, there were no observable differences and no damage was found in the healthy urothelium of both mice (Figure 4C,D, blue arrows).

### 2.6 In Vivo PDT Efficacy

**Figure 5:**
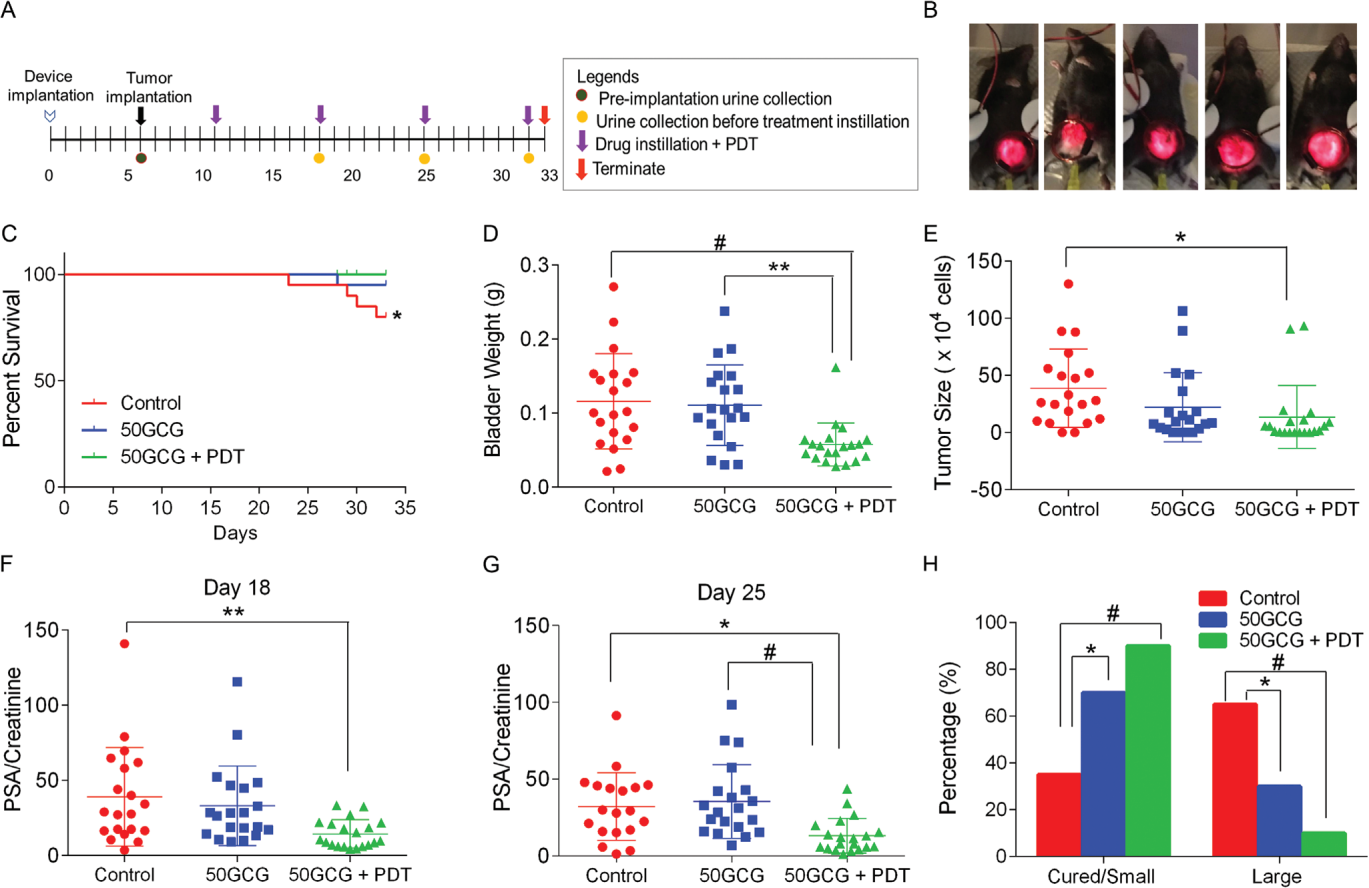
In vivo efficacy of 50GCG combined with wireless PDT in a mouse orthotopic bladder cancer model. A) Schedule of in vivo efficacy experiment. B) Images of activation of 5 implanted devices with wireless powering at input power 121 mW. C) Kaplan–Meier analysis of disease‐specific survival of the mice in the efficacy experiment. * Log‐rank test for trend significance *p* < 0.05. D) Weight of harvested bladders after experiment termination. E) Tumor size as indicated by the number of MB49‐PSA cells in each bladder tissue. Free PSA levels in the urine on F) Day 18 and G) Day 25 of the experiment schedule. H) Percentage of mice in each group with small/cured or large tumor sizes. Tumor sizes were defined as small/cured (<20 × 10^4^ cells) or large (>20 × 10^4^ cells) according to the descriptive statistics of control group mice (Table S2, Supporting Information). The experiment was done twice (*n* = 20). Data were represented as mean ± SD. * *p* < 0.05, ** *p* < 0.01, # *p* < 0.005.

We devised a 33‐d long once‐weekly treatment regimen to demonstrate the feasibility of using the implanted device for an extended schedule (**Figure** **5**A) compared to our previous work.^[^ ^18^ ^]^ An initial experiment was performed (*n* = 5) to detect any nanocarrier or device‐related complications and to compare targeted versus nontargeted photodynamic‐chemotherapy effects in a low‐powered study (Figure S16, Supporting Information). The cure rate data from the small study (Table S3, Supporting Information) provided a strong assumption for power analysis of proportions calculations to plan for a larger PDT efficacy study. However, due to the large number of mice needed to provide significant data for targeted versus nontargeted photodynamic chemotherapy (745 mice per group), the larger study focused on investigating the efficacy of targeted photodynamic chemotherapy only. All the mice in the targeted photodynamic‐chemotherapy group (50GCG + PDT) received four complete doses of PDT except for two mice that died early on Days 28 and 29 (Figure 5B). For these two mice, advanced disease was initially suspected, but the bladders looked small, and no PSA gene (which serves as a tumor marker) was detected during qPCR analysis. The probable cause of death was deduced to be an infection as the urine voided by the mice was cloudy. There were four early terminations in the control and one early termination in targeted chemotherapy‐only (50GCG) group. For these groups, the bladder samples were positive for the PSA gene, indicating tumor presence. No significant difference was found in the overall survival between all groups (Figure S14A, Supporting Information). However, when the absence of tumors in the mice that died early in the targeted photodynamic‐chemotherapy group was factored in, the trend for disease‐specific survival was significant (*p* = 0.0268, Figure 5C).

The bladder and draining lymph nodes (DLN) weights were also evaluated since bladder weight can indicate tumor burden, while an increase in DLN weight may signal an ongoing immune response to the tumor or treatment. Mean bladder weight from the targeted photodynamic‐chemotherapy group was reduced compared to control (*p* = 0.0021) and targeted chemotherapy‐alone (*p* = 0.0055, Figure 5D). Although the targeted photodynamic‐chemotherapy group recorded the lowest mean DLN weight, the differences were not significant (Figure S14B, Supporting Information). No significant differences were found in the bladder PSA gene expression using relative quantification methods by qPCR (Figure S14C, Supporting Information). Hence, a quantifiable assay was devised to measure tumor size by generating a calibration curve from ascending amounts of MB49‐PSA cells using their delta Ct (Cycle threshold) mean values (Figure S15, Supporting Information). A significant decrease in tumor size was observed in the targeted photodynamic‐chemotherapy group only (*p* = 0.0374, Figure 5E).

Urine was collected on Days 18, 25, and 32 to monitor tumor growth via free PSA (fPSA) ELISA and to detect successful tumor implantation in each mouse based on preimplantation values on Day 5. The tumor implantation rate for this experiment was 100%. A significant reduction of the fPSA levels in the targeted photodynamic‐chemotherapy group can be seen one week after the first treatment dose on Day 18 (*p* = 0.0082, Figure 5F). The significant decrease continued on Day 25, when the mice had completed two doses. Urinary PSA levels of the targeted photodynamic‐chemotherapy group were significantly lower than control (*p* = 0.0134) and targeted chemotherapy‐only group (*p* = 0.0024, Figure 5G) on Day 25. On Day 32, the significance was not observed because three mice from the control group were terminated early, and the tumor size increased for one mouse in the targeted photodynamic‐chemotherapy group (Figure S14D, Supporting Information).

Cure rates for the targeted chemotherapy‐only and targeted photodynamic‐chemotherapy groups were 15% and 35%, respectively. The control group has two spontaneous cures (10%), which is expected with the orthotopic model.^[^ ^22^ ^]^ Besides curing tumors, controlling the tumor burden is another important measure of treatment efficacy that constitutes treatment response, given the mainly adjuvant role of chemotherapy in postresected NMIBC. Hence, we analyzed the tumor burden in a binary approach with two categories: cured/small tumors and large tumors. The ranges of the tumor size categories were based on half the mean tumor size of the control group (Table S2, Supporting Information). Hence, cured/small tumors range was defined as <20 × 10^4^ cells and large tumors are >20 × 10^4^ cells. Compared to the control (35%), the targeted photodynamic‐chemotherapy group had the highest proportion of mice with cured and reduced tumor sizes at 90% (*p* = 0.001), while the targeted chemotherapy‐only group showed 70% treatment efficacy (*p* = 0.03, Figure 5H). Hence, both targeted chemotherapy‐only and targeted photodynamic‐chemotherapy treatments demonstrated significant treatment responses in mice.

## 3 Discussion

In this study, we demonstrated our wirelessly powered and targeted photodynamic‐chemotherapy strategy as a potential treatment modality for NMIBC. The key elements of our strategy are glycosylated micellar nanocarriers with coloaded Ce6 and GemE for selective intravesical drug delivery to bladder tumors, and implantable millimeter‐sized wireless LED device powered by radio frequency (RF) for programmable light delivery. As a proof of concept, we tested the efficacy of this novel approach in vivo in an orthotopic murine model of bladder cancer. The implantability of the light‐emitting device enabled an extended schedule where multiple PDT can be performed to improve treatment outcomes. To our knowledge, this is the first demonstration of in vivo photodynamic‐chemotherapy to achieve an enhanced therapeutic effect in an orthotopic cancer model using an implantable and wirelessly powered LED device.

Steering intravesical drug delivery specifically into tumor tissues is a considerable clinical interest. However, targeting bladder tumors using surface markers is challenging as the disease lacks established specific receptors for targeted drug delivery to all bladder cancer types, stages, and grades.^[^ ^23^ ^]^ Hence, we capitalized on GLUTs as an alternative target as it is overexpressed in urothelial cancers, making glucose a potentially effective and economical targeting agent.^[^ ^24^ ^]^ However, little work of applying glycosylated carriers to target and treat bladder tumors has been reported. The PS‐loaded glycosylated nanocarriers displayed enhanced targeting efficacy in bladder cancer cell lines, leading to increased PDT induced cytotoxic effect. Furthermore, we validated the effectiveness of the PS‐loaded glycosylated nanocarriers in directing selective PS accumulation in orthotopically implanted tumors, which is a significant step in potentially reducing AEs that have impeded prevalent PDT applications in clinical settings. In addition, our results showed that the chemotherapeutic outcome of GemE can be enhanced under light irradiation by the PCI effect, therefore achieving treatment synergy.^[^ ^11^ ^]^

Miniaturizing the wireless LED device was a critical step in ensuring implantability. Though the device could not be positioned within the bladder lumen in mice due to size constraints, it is suitable for grafting in human bladders via minimally invasive surgical procedures. However, the device must be further miniaturized in the long term to achieve cystoscopy‐guided implantation compatibility.^[^ ^25^ ^]^ Nevertheless, the device showed excellent in vivo survival in the 33‐d experiment schedule, supplying four once‐weekly light doses for PDT. This is, by far, the most extended in vivo schedule demonstrated using implantable wireless photonics for cancer PDT.^[^ ^18^, ^26^ ^]^ Bogaards et al. established PDT regimens with more light doses (5–14 doses), but the light supply was powered using electrical wires or external batteries.^[^ ^27^ ^]^ Our device can remain functional longer than the current study period, allowing real‐time therapy customization to patients' responses. Furthermore, the light dose supplied to the bladder is significantly lower (1.8 J cm^−2^) than the light doses used for conventional PDT in bladder cancer therapy, which uses doses of >10 J cm^−2^.^[^ ^28^ ^]^ The low light dose used in our study demonstrated the positive influence of our targeted photodynamic‐chemotherapy regimen as low irradiance is sufficient to activate the boosted accumulated PS in the tumor tissues delivered by the glycosylated nanocarriers. Using low light doses is advantageous as it will ensure minimal tissue temperature increase, thus protecting the tissues from thermal damage. The use of implantable wireless LEDs confers another advantage whereby multiple wavelengths can be supplied to match the absorption peaks of regularly used PSs. Both 405 and 660 nm LEDs were fabricated on the device, supplying light to the bladders at radiant power of 1 mW (405 nm) and 0.5 mW (660 nm), respectively, ensuring optimal Ce6 activation.

Treating NMIBC with intravesical drugs is a challenge due to the limited retention time in the bladder, requiring multiple weekly treatment doses to achieve clinical response. However, our targeted photodynamic‐chemotherapy approach demonstrated effective tumor burden reduction after only one dose based on urinary PSA levels, and the effect remained durable after the second dose. In contrast, standard intravesical BCG treatment requires at least 4–6 weekly doses to mount the necessary immune response for therapeutic efficacy.^[^ ^29^ ^]^ Both targeted approaches (chemotherapy‐only and combined therapy) also successfully cured and controlled bladder tumor burden, with the photodynamic‐chemotherapy regimen displaying enhanced treatment response than chemotherapy‐alone. The aims of achieving tumor burden reduction or cure were used as appropriate clinical outcomes, based on the fact that the current clinical role of intravesical immunotherapy and chemotherapies are not to be directly curative but rather adjuvant in nature (following resection of visible tumors), where reduction of residual tumor burden or recurrence is the intent. Our study is significant as we showed that a wirelessly powered, low‐irradiance, and low‐dose photodynamic chemotherapy with enhanced therapeutic effect could be successfully applied to eradicate orthotopically implanted bladder tumors.

Our approach can be extended to treat deep‐seated tumors since the implantable LED device can overcome the limiting light penetration issues associated with surface‐applied light sources. It can also be further adapted for other clinical applications that require targeted delivery to diseased tissues such as photothermal therapy and optogenetics, moving the possibility of practical and successful clinical applications several strides forward.

## 4 Experimental Section

### Synthesis of Glucosamine Conjugated DSPE‐PEG (DSPE‐PEG‐Glu)

Glucosamine was conjugated to DSPE‐PEG according to the protocol reported by Annapragada et al.^[^ ^30^ ^]^ with minor modifications. Briefly, 20 mg 1,2‐distearoyl‐sn‐glycero‐3‐phosphoethanolamine‐*N*‐[carboxy(polyethylene glycol)‐2000] (DSPE‐PEG(2000)‐COOH) was dissolved in 1 mL dimethylformamide (DMF), and then 4.14 mg (3 mol equivalents) 1‐ethyl‐3‐(3‐dimethylaminopropyl)carbodiimide (EDC) and 2.5 mg *N*‐hydroxysuccinimide (NHS, 3 mol equivalents) dissolved in 0.2 mL (DMF) respectively were added sequentially to activate the carboxylic group of DSPE‐PEG(2000)‐COOH under magnetic stirring at 450 rpm overnight. Deprotonated glucosamine was prepared by dissolving 5 mg glucosamine in 0.1 mL Milli Q water which was then mixed with 30 µL triethylamine (TEA) in 1 mL DMF. This solution was then added dropwise into the above‐mentioned DSPE‐PEG(2000)‐COOH solution after EDC/NHS activation to react for 48 h under 450 rpm magnetic stirring. The product was then purified by dialysis in a 1000 Da dialysis tube against Milli Q water for 24 h with frequent water changes. Finally, the product was lyophilized (denoted as DSPE‐PEG‐Glu), and its structure was characterized by ^1^H NMR with a 400 MHz Bruker DMX400 spectrometer using chloroform‐d (CDCl_3_) as solvent.

### Synthesis and Characterization of Ce6‐ and Drug‐Loaded Carriers

The details of the methods are given in the Experimental Section in Supporting Information.

### Device Assembly

The wireless PDT device comprises an eight‐turn spiral coil for receiving magnetic field, a half‐wave voltage doubler, a rectifier for alternating current to direct current conversion, and two light‐emitting diodes (LEDs). The customized two‐sided printed circuit board (PCB, fabricated through Interhorizon Corporation Pte Ltd, China) was designed for reducing the size and increasing the number of turns (Figure S11A,B, Supporting Information). Figure S11 in the Supporting Information presents each side of the PCB. The serially connected 660 nm LED (SML‐LX0603SRW‐TR, Lumex, USA) and 405 nm LED (SM0603UV‐400, Bivar Inc, USA) were placed on the top side (Figure S11C, Supporting Information). The capacitors (100‐pF capacitor (06031A101JAT2A, AVX Corporation, USA), 220‐pF capacitor (C0603C221K1RACTU, KEMET, USA), 27‐pF capacitor (GCM0335C1H270JA16D, Murata Electronics, Japan) and Schottky diodes (BAT2402LSE6327XTSA1CT‐ND, Infineon Technologies, Germany) consisting of the half‐wave voltage doubler were placed on the bottom side (Figure S11D, Supporting Information). All the components were mounted using micro‐soldering (NAE‐2A, JBC, USA) under a microscope (SZ61, Olympus, Japan) with lead‐free soldering materials (SMD291SNL10, ChipQuik, USA). The electronics were coated by applying epoxy to prevent mechanical and moisture‐induced damage (Figure S11E,F, Supporting Information). Multiple devices were encapsulated in a 3D printed mold by pouring PDMS, degassing in a vacuum chamber for 30 min, and curing in a forced convection oven (Esco Isotherm, SG) at 70 °C for 1 h. The encapsulated devices were removed from the mold, and the remaining cracks on the surface were filled with rapid curing, biocompatible silicone (WPI, Kwik‐Sil, UK) (Figure S11G,H, Supporting Information).

### In Vivo Efficacy Evaluation in an Orthotopic Murine Bladder Cancer Model

The animal protocols in this study were approved by the Institutional Animal Care and Use Committee, National University of Singapore (R17‐1435). Surgical device insertion was performed, and the wounds were completely healed before tumor implantation (Figure 4A). C57BL/6 mice (6–8 weeks old, InVivos, Singapore) bladders were accessed intravesically using a 24 G plastic cannula following anesthesia with an intraperitoneal injection of 75 mg kg^−1^ ketamine and 1 mg kg^−1^ medetomidine. Intrabladder solutions were administered in 50 µL volume unless stated otherwise. First, the bladders were treated with PLL for 20 min followed by 1 h with MB49‐PSA cells (2 × 10^6^ cells mL^−1^, in blank DMEM) immediately after PLL voidance. The MB49‐PSA cell line was engineered to secrete the human PSA gene, which can be used as a marker for tumor growth by detecting it in urine or bladder tissues.^[^ ^31^ ^]^ Intrabladder treatments with 2 h dwell time commenced five days after tumor implantation followed by bladder washings with 0.1 mL of saline. Control mice were given saline while targeted chemotherapy‐only mice were given 50GCG (0.2 mg mL^−1^, 16 × 10^−6^ M Ce6 with 8 × 10^−6^ M GemE). The combined photodynamic‐chemotherapy group was given 50GCG followed by wireless irradiation at 121 mW for 30 min, providing light at a total dose of 1.8 J cm^−2^ to the mouse bladder. Atipamezole (0.1 mg kg^−1^) solution was administered after treatment completion. The treatment was repeated once weekly for a total of four doses. Mice were terminated one day after the last treatment dose via CO_2_ asphyxiation followed by cervical dislocation. PSA gene detection in the bladder tissues, urinary free PSA and creatinine measurements were done as previously described.^[^ ^32^ ^]^ Detailed methods can be found in Additional Methods in the Supporting Information.

### Statistical Analysis

Comparisons of means between multiple groups were performed using one‐way ANOVA with a Bonferroni post‐hoc test using GraphPad v6 software (GraphPad, SanDiego, CA, USA). Differences between means were considered statistically significant when the two‐tailed test *p*‐value was <0.05. Representation of the *p*‐value was indicated in each respective figure. Kaplan–Meier survival analysis was plotted with GraphPad software, and curve comparisons were determined with inbuilt log‐rank tests. Determination of sample sizes was performed using G‐power open‐sourced software with Fisher's exact test (v3.1). The assumptions obtained from the small initial in vivo testing were used to determine the number of mice needed to show significance at 5%, power at 80%, and a two‐sided test. Proportion analysis of tumor sizes was performed with IBM SPSS Statistics v 25 using the Chi‐square test.

## Conflict of Interest

The authors declare no conflict of interest.

## Author Contributions

Conceptualization: Y.Z., K.G.N., J.S.H., Methodology: Y.Z., K.G.N., J.S.H., R.M., K.E., E.C., J.N.R.B., Formal analysis: B.S., J.N.R.B., H.J.K., Investigation: B.S., J.N.R.B., H.J.K., Writing – Original Draft: B.S., J.N.R.B., H.J.K., Writing – Review and Editing: Y.Z., K.G.N., R.M., Visualization: Y.Z., K.G.N., Supervision: Y.Z., Project administration: Y.Z., Funding acquisition: Y.Z.

## Supporting information

Supporting information

## Data Availability

The data that support the findings of this study are available from the corresponding author upon reasonable request.
