# Supplementary material for: Wirelessly Activated Nanotherapeutics for In Vivo Programmable Photodynamic‐Chemotherapy of Orthotopic Bladder Cancer
Source: Adv Sci (Weinh). 2022 Apr 7;9(16):2200731. doi: 10.1002/advs.202200731 (PMC9165499; doi:10.1002/advs.202200731)
Supplement: Supplementary file 1 — Supporting information [file ADVS-9-2200731-s001.pdf]

ADVANCED SCIENCE
Open Access

Supporting Information

for *Adv. Sci.*, DOI 10.1002/advs.202200731

Wirelessly Activated Nanotherapeutics for In Vivo Programmable Photodynamic-Chemotherapy of Orthotopic Bladder Cancer

*Bowen Sun, Juwita Norasmara Bte Rahmat, Han Joon Kim, Ratha Mahendran, Kesavan Esuvaranathan, Edmund Chiong, John S. Ho, Koon Gee Neoh and Yong Zhang**

Supporting Information

Wirelessly activated nanotherapeutics for *in vivo* programmable photodynamic-chemotherapy of orthotopic bladder cancer

*Bowen Sun, Juwita Norasmara Bte Rahmat, Han Joon Kim, Ratha Mahendran, Kesavan Esuvaranathan, Edmund Chiong, John S. Ho, Koon Gee Neoh, and Yong Zhang**

B.Sun and Prof. K.G. Neoh
Department of Chemical and Biomolecular Engineering, Faculty of Engineering, National University of Singapore, Singapore 117585, Singapore

Dr. H.J. Kim and Dr. J.S. Ho
Department of Electrical and Computer Engineering, Faculty of Engineering, National University of Singapore, Singapore 117583

Dr. J.S. Ho
Institute for Health Innovation and Technology, National University of Singapore, Singapore 119276
The N.1 Institute for Health, National University of Singapore, Singapore 117456

Dr. R. Mahendran, Prof. K. Esuvaranathan, and Prof. E. Chiong
Department of Surgery, Yong Loo Lin School of Medicine, National University of Singapore, Singapore 119228

Prof. K. Esuvaranathan and Prof. E. Chiong
Department of Urology, National University Health System, Singapore 119228

Dr. J.N.B Rahmat, and Prof. Y. Zhang
Department of Biomedical Engineering, Faculty of Engineering, National University of Singapore, Singapore 117583
B.Sun and J.N.B Rahmat contributed equally to this work

E-mail: biezy@nus.edu.sg

Additional Methods

Materials

Chlorin e6 (Ce6) and gemcitabine elaidate (GemE) were purchased from Frontier Scientific (Frontier Scientific Inc., US). Fetal bovine serum (FBS), Dulbecco's Modified Eagle's Medium (DMEM) were purchased from GE (GE Inc., US). Penicillin-streptomycin (10,000 U ml^{-1}), LysoTracker Green DND-26, carboxyfluorescein succinimidyl ester (CFSE) dye, and LIVE/DEAD Viability/Cytotoxicity Kit for mammalian cells were purchased from Thermo Fisher Scientific (Thermo Fisher Scientific Inc., US.). All the other chemicals and solvents were purchased from Sigma-Aldrich (US).

Preparation of glycosylated micellar carriers loaded with Ce6

Mixtures of 1,2-distearoyl-sn-glycero-3-phosphoethanolamine-*N*-[methoxy(polyethylene glycol)-2000] (DSPE-PEG(2000)-OMe) and DSPE-PEG-Glu of a total weight of 10 mg but with varying weight ratios were used to prepare carriers of different degrees of glycosylation. The mixture was put in a 20 ml glass vial and dissolved in 0.5 ml dimethyl sulfoxide (DMSO) under magnetic stirring. Ce6 (50 µl) dissolved in DMSO solution (12 mg ml^{-1}) was added and stirred for 30 min, followed by rapid addition of 2 ml Milli Q water under vigorous stirring (1,000 rpm). The vial was then sonicated in an ice bath in a bath-type sonicator for 20 min. Finally, the solution was transferred to a 1,000 Da dialysis tube to dialyze against Milli Q water overnight. The product was further purified by passing through a 0.22 µm nylon filter. The products were denoted as 0GC, 10GC, 20GC, 30GC, 50GC, 70GC, and 100GC, with the numbers indicating the weight percent of DSPE-PEG-Glu in the phospholipid mixture used in the preparation of the

carriers. The samples were kept in deionized water and stored at 4 °C to be used as stock in subsequent experiments.

Co-loading Ce6 and GemE in glycosylated carriers

The protocol of co-loading Ce6 and GemE was the same as that for loading Ce6 described above, except the weight percent of DSPE-PEG-Glu used in the synthesis of the carriers was fixed at either 0 or 50%. Briefly, 50 µl Ce6 DMSO solution (12 mg ml^{-1}) and 50 µl GemE DMSO solution (5.7 mg ml^{-1}) were added to the lipid-PEG DMSO solution before the addition of water. The samples were denoted as 50GCG and 0GCG, depending on whether DSPE-PEG-Glu was added in the carrier (at 50%) or not.

Characterization of Ce6- and drug-loaded carriers

The zeta potential of the nanocarriers was determined using a Malvern ZS Zetasizer Nanoseries (Malvern Instruments Ltd., UK). The particle sizes and morphologies of the nanoparticles were assessed by transmission electron ~~microscopes~~ microscopy (TEM) and high-resolution transmission electron ~~microscopes~~ microscopy (HRTEM) using a field emission transmission electron ~~microscopy~~ microscope (Model JEOL-2010-F from JEOL Ltd, Tokyo, Japan). The fluorescence spectra of 50GCG, 0GCG, 50GC, 0GC, or free Ce6 in 1×phosphate-buffered saline (PBS) solution with 0.1% DMSO with Ce6 concentration adjusted to 5 µM was measured using a Shimadzu RF-6000 spectrofluorophotometer (Shimadzu Ltd., Japan) (excitation wavelength (Ex) = 405 nm).

To determine Ce6 and GemE loading in the carriers, these carriers were first lyophilized and redissolved in DMSO. The concentration of Ce6 was determined using a Shimadzu UV-2600

UV-vis spectrophotometer (Shimadzu Ltd., Japan) and a standard curve of free Ce6 concentrations in DMSO. The amount of loaded GemE was determined using high-performance liquid chromatography (HPLC) with a C18 reverse phase column and a linear gradient elution from 15% acetonitrile in water to 80% acetonitrile in water over 30 min at 1 ml min^{-1} flow rate. UV absorption by the HPLC eluent at 280 nm was recorded. A standard curve of free GemE in DMSO was generated using the same protocol. The loading (LE) and encapsulation efficiencies (EE) were calculated as follows:

$$\mathrm{LE} = \frac{weight\ of\ Ce6\ (or\ GemE)}{weight\ of\ drug{-}loaded\ carriers} \times 100\%$$

$$\mathrm{EE} = \frac{weight\ of\ Ce6\ (or\ GemE)\ in\ carriers}{weight\ of\ Ce6\ (or\ GemE)\ added} \times 100\%$$

To monitor the release of Ce6 and GemE from the glycosylated and non-glycosylated carriers, 1 ml 50GCG or 0GCG dispersed in PBS (Ce6 concentration = 40 µM for all samples) was packed in 2,000 Da dialysis tubes (MWCO = 2,000 Da). The dialysis tubes were then immersed in 20 ml artificial urine [1], which was gently shaken at 150 rpm and maintained at 37 °C. At predetermined times, 4 ml of the release medium was sampled and lyophilized, and a similar volume of artificial urine was added back to maintain a constant volume. DMSO (1 ml) was then added to the lyophilized release medium followed by centrifugation for 5 min at 5,000 rpm, the precipitate (comprising salts from artificial urine that were not soluble in DMSO) was discarded and the concentrations of Ce6 and GemE were determined by HPLC with a C18 reverse-phase column using the same protocols as described above.

Cell culture and *in vitro* cellular uptake of free Ce6, Ce6-loaded, and Ce6 and GemE co-loaded nanocarriers

Human urothelial carcinoma cell line, UMUC3 purchased from ATCC was cultured in DMEM supplemented with 10% heat-inactivated FBS and 1% penicillin-streptomycin. Mouse bladder carcinoma cell line, MB49 (a kind gift from Dr. Timothy Ratliff, Purdue University), was cultured in RPMI-1640 supplemented with 10% FBS and 1% penicillin-streptomycin. The cultures were maintained at 37 °C in a humidified 5% CO_2 atmosphere. All the chemicals and samples used to treat cells were sterilized by passing through 0.22 µm nylon syringe filters.

To measure cellular uptake of free Ce6 or Ce6-loaded nanocarriers, the cells were seeded in a 24-well plate (3×10^5 cells per well) and incubated at 37 °C overnight. The medium was then replaced with 0.5 ml fresh medium containing either free Ce6, Ce6-loaded, or Ce6 and GemE co-loaded nanocarriers with 0.1% DMSO (5 µM Ce6 for all samples). After incubation for 2 h, the cells were washed with 1×PBS three times, trypsinized, and resuspended in 1×PBS containing 2% FBS for fluorescence-activated cell sorting (FACS) flow cytometry analysis with a CytoFLEX LX flow cytometer (Beckman Coulter Ltd., US).

The cellular uptake of 50GCG, 0GCG, and free Ce6 was also assessed using confocal laser scanning microscopy (CLSM, Nikon, Eclipse Ci, Nikon, Japan) equipped with a 40× water-immersion objective lens and appropriate filters. The UMUC3 cells were incubated on glass-bottom four-well plates overnight (5×10^4 cells per well). The medium was then replaced with 0.5 ml fresh medium containing free Ce6, 0GCG, or 50GCG (5 µM Ce6). After incubation at 37 °C for 2 h, the cells were fixed with 4% paraformaldehyde and washed twice with 1×PBS. The

nuclei of the cells were stained with 4′,6-diamidino-2-phenylindole (DAPI) before the cells were visualized with CLSM.

***In vivo* targeting assay of glycosylated carriers against MB49 tumors**

MB49-PSA cell line was labeled with 10 µM of CFSE dye according to the manufacturer's instructions. All intrabladder instillation was done in 50 µl unless stated otherwise. Poly-L-lysine (PLL, 0.01%, Sigma, MO, USA) was instilled in the bladder for 20 min followed by complete voidance. The labeled cells (2×10^6 cells ml^{-1}) were left to dwell in the bladder for 1 h. Three days after implantation, 0.2 mg ml^{-1} non-targeting (0GC) or targeting (50GC) formulation with Ce6 payload (16 µM) was instilled in the bladder for 2 h. Bladders were washed with 0.1 ml of 1×PBS before termination. Bladders were harvested from mice carcasses and embedded in OCT cryomedia (Thermo Fisher, MA, USA). Tissue sections (6-8 µm) were cut using a Leica cryostat (Wetzlar, Germany) and positioned on polysilane slides. The slides were washed 3× with 1×PBS, stained with 1 µg ml^{-1} DAPI, and the fluorescence signals were detected using a Zeiss LSM710 confocal microscope (Oberkochen, Germany).

Measurement of intracellular ROS generation of UMUC3 cells

To detect the intracellular generation of ROS from 50GCG, 0GCG, and free Ce6, UMUC3 cells were seeded in glass-bottom 8-well plates (3×10^4 cells per well) and incubated overnight. The medium was then replaced with 0.5 ml medium containing 50GCG, 0GCG, or free Ce6 (5 µM Ce6 for all samples) and incubated for 2 h. The cells were washed twice with 1×PBS, and the ROS detector 2',7'-dichlorofluorescein diacetate (DCFH-DA) (10 µM) dissolved in 0.5 ml serum-free medium was added. After further incubation for another 30 min at 37 °C, the cells were washed with 1×PBS once and were then irradiated with 660 nm laser (Diode-pumped solid-

state laser, Photonitech (Asia) Pte Ltd. Singapore) at 250 mW cm^{-2} for 1 min. Cells that were not irradiated served as the control group. DCFH-DA, after diffusion into cells, will be hydrolyzed to form non-fluorescent DCFH. The reaction of DCFH with ROS forms 2',7'-dichlorofluorescein (DCF), which fluoresces at 525 nm under excitation at 488 nm. The fluorescent DCF was detected using a Nikon Eclipse Ti-U fluorescence microscope (Nikon, Japan) equipped with a 20× objective lens ((Ex) = 488 nm, Emission wavelength (Em) = 525 nm).

***In vitro* photo- and chemo-toxicity assays of free Ce6 and nanocarriers loaded with Ce6 or Ce6 and GemE**

UMUC3 or MB49 cells were seeded on 96-well plates (5 × 10^{3} cells per well) and incubated overnight at 37 °C. The medium was then replaced with 0.1 ml fresh medium with 0.1% DMSO containing free Ce6 or carriers loaded with Ce6 only or Ce6 and GemE at different concentrations. After incubation for 2 h, the cells were washed twice with 1×PBS and then irradiated by the 660 nm laser (250 mW cm^{-2}) for 5 min except for the control groups. All groups were then incubated for 3 days, and 3-(4,5-dimethylthiazol-2-yl)-2,5-diphenyltetrazolium bromide (MTT) assay was conducted. Briefly, the medium was replaced with fresh medium containing 0.5 mg ml^{-1} MTT and incubated for another 4 h. The medium was then removed, and formazan crystals at the bottom of each well were dissolved with 0.1 ml of DMSO and shaken for 10 min until all the crystals were dissolved. The absorption at 595 nm was measured with a microplate reader, and the results were expressed as percentages relative to the absorbance measured with the control sample (cells incubated in medium only).

The viability of the cells after treatment with 50GCG or 0GCG and with or without laser irradiation was also assessed using live/dead staining. UMUC3 cells (3 × 10^{4} per well) were

seeded on a 96-well plate and cultured at 37 ℃ overnight. The medium was then replaced with 0.1 ml fresh medium containing 50GCG or 0GCG with Ce6 concentration of 5 µM. After 2 h incubation, the cells were washed with 1×PBS once, and 0.1 ml of fresh DMEM was added to each well. Some groups of cells were irradiated with the 660 nm laser (250 mW cm^{-2}) for 3 min, while those cells that were not irradiated served as the non-irradiated control group. Then, all the cells were stained with 2 µM calcein-AM and 5 µM ethidium homodimer-1 in 0.1 ml medium for 30 min at 37 ℃. Finally, the cells were washed with 1×PBS and immediately observed with a Nikon Eclipse Ti-U fluorescence microscope (Nikon, Japan) equipped with a 10× objective lens. (calcein-AM imaging: Ex = 488 nm, Em = 525 nm; ethidium homodimer-1 imaging: Ex = 580 nm, Em = 630 nm).

Evaluation of the combined effects of Ce6 and GemE co-loaded in glycosylated carriers.

The combined effects of PDT with Ce6 and GemE on the cells were evaluated using the equations reported by Chou et al.[2]:

$$D_X = D_m [\frac{Fa}{1-Fa}]^{\frac{1}{m}}$$

$$\mathrm{CI} = \frac{(D)1}{(D_X)1} + \frac{(D)2}{(D_X)2}$$

(Treatment 1 = Ce6 + 660 nm irradiation, Treatment 2 = GemE)

D denotes the dose of Ce6 (1) or GemE (2) when used in combination photodynamic-chemotherapy, Fa denotes the fraction of cells affected by the respective dose D, D_m denotes the dose required to produce the median effect, i.e., IC_{50}, and m is a Hill-type coefficient signifying the sigmoidicity of the dose-effect curve. D_X was calculated from the results obtained when the

cells were subjected to either PDT with Ce6 (1) or chemotherapy with GemE (2). The combination index (CI) values were calculated from the above equations using Compusyn software written in BASIC. The effects of the loaded Ce6 subjected to laser irradiation and GemE could either be mutually nonexclusive or mutually exclusive, with CI > 1 indicating an antagonistic effect of PDT with Ce6 and GemE, CI < 1 indicating a synergistic effect of Ce6 and GemE, and CI = 1 indicating an additive effect.

To obtain a better understanding of the combined effects of PDT and chemotherapy from the Ce6 and GemE co-loaded in the nanocarrier, the lysosomes of UMUC3 cells were tracked after being subjected to chemotherapy with or without PDT. UMUC3 cells were seeded on 8-well glass-bottom plates (2×10^4 cells per well) and incubated in a humidified 5% CO_2 atmosphere at 37 °C overnight. The medium was then replaced with 0.1 ml fresh medium or 0.1 ml fresh medium containing 50GCG (5 µM Ce6). After incubation for 2 h, the cells were washed with 1×PBS twice. Staining of the lysosomes was carried out by incubating the cells in 0.1 ml fresh medium containing 100 nM of LysoTracker green DND-26 for 90 min followed by washing with 1×PBS twice. Then, some groups were irradiated with 660 nm laser (250 mW cm^{-2}) for 30 s, while non-irradiated cells served as the control group. The nuclei of the cells were stained with DAPI for 5 min. The cells were then fixed with 4% paraformaldehyde 1×PBS solution for 15 min and washed with 1×PBS twice. Finally, the cells were imaged using a Nikon A1R CLSM (Nikon, Japan) equipped with a 100× oil immersion objective lens and appropriate filters.

Device characterization

In the benchtop test, the radiant power of the device was evaluated using an optical power meter (2935-C, Newport, USA) or estimated with a rectified current of the device and datasheet of each

LED. The RF input power was controlled by a signal generator (Model 835, Berkeley Nucleonic Corp., USA). To investigate the change in radiant power with distance, the device was first placed at the center of the transmitter coil (4 turn coil with 10 mm radius), which was fixed on a translational stage. The transmitter coil was then moved vertically away in a constant direction from the wireless device, and the change in current with increasing distance was recorded from the multimeter (Fluke 289, Fluke, USA).

***In vitro* PDT of MD49 cells with the wireless device**

MB49 cells (1.5× 10^4) were plated in 96 well plates overnight. The next day, they were treated with free Ce6 or 50GCG for 2 hours and washed twice with 1× PBS. Fresh media (0.1 ml) was added to each well and irradiation was performed for 30 minutes in the dark with dual micro-LED device (405 and 660 nm) wirelessly activated with 100 mW input power. The cells were incubated at 37 °C for a further 24 hours before viability measurement by adding 20 μL of MTS reagent (Promega, Madison, WI, USA) into each well and incubated for 4 hours at 37°C incubator. Raw absorbance data at 490 nm and 630 nm was obtained using a multiplate reader (Biotek, Synergy H1, Winooski, VT, USA). The results were analysed by subtracting the OD630 nm measurements from OD490 nm raw data and calculating the percentage of cells in each sample groups referenced with respective controls.

Implantation of the wireless device in mice

Mice were anesthetized (isoflurane: 4% induction and 2% maintenance, with 100% O_2 as a carrier) and prepared for surgery. Under anesthesia, a heating pad underneath the mice maintains the body temperature at 37 °C. An incision (~8 mm) was made on the linea alba close to the pelvic region on the abdomen. The bladder of the mouse was inflated by instilling saline (0.1 ml)

via a 24 G catheter. The inflated bladder can be visualized over the thin abdominal muscle tissue and serves as a guide for positioning the device. The device was secured on the muscle tissue using the three holes provided on the device encapsulation with sutures (5/0 reverse cut needle undyed absorbable glyconate monofilament, BBraun, Melsungen, Germany). The wound was then closed using a horizontal mattress suture technique followed by butyl cyanoacrylate adhesive (Vetbond, MN, USA) applied to the sealed wound. A wound clip was used to secure the wound apposition. The mice were left to recover in a separate cage with a heating pad and monitored until conscious, mobile, and responsive before returning to the home cage. Post-operative care (5 mg kg^{-1} carprofen and 0.1 mg kg^{-1} buprenorphine) was administered daily for 5 days. The wound was monitored daily until complete recovery.

***In vivo* SOSG assay, thermal effects of RF exposure, and TUNEL assay**

Mice with implanted devices for at least 11 days were anesthetized (intraperitoneal injection of 75 mg kg^{-1} ketamine and 1 mg kg^{-1} medetomidine) before the assay. The glycosylated formulation (0.2 mg ml^{-1} 50GC, 16 µM Ce6) containing 10 µM SOSG solution was instilled in the bladder at 0.1 ml volume. Irradiation with the wireless light source was performed at 10, 20, and 30 min. Fresh 50GC test solution from the same stock was instilled in the bladder at every time point. The tissue temperature probe, connected to a multimeter (Fluke), was placed on the abdominal tissue closest to the device via an incision made on the skin for temperature measurements during the irradiation period. Two wireless activation settings were tested (input power 121 mW and 245mW). Mice were terminated after the assay and sent for CT scan (Micro-CT Quantum FX, performed by a radiographer in Comparative Medicine, NUS). Bladders from mice implanted with tumors and treated with 50GCG and irradiation were harvested and

embedded in OCT cryomedia. Bladder tissue sections (6-8 µm) were used for TUNEL assay according to the manufacturer's protocol (Thermo Fisher, USA). The fluorescence was detected and imaged with the Zeiss LSM710 confocal microscope.

Haematoxylin and Eosin Staining

Harvested tissues were fixed immediately in 10 % neutral buffered formalin for at least 24 hours. The tissues were then sent for processing and paraffin embedding at the NUS Medicine Core for Microscopy and Analysis facility. Tissue sections were cut in 8-10 µm sections and fixed on polysilane coated slides (Thermo Fisher, USA). The slides were stained with hematoxylin and eosin according to the following protocol: Paraffin wax was removed by xylene immersion for 10 minutes followed by a solution of 50% xylene and 50% ethanol for 2 minutes. The tissues were dehydrated by sequentially immersing the slides in 100% ethanol for 10 minutes, 95% ethanol for 5 minutes, and 70% ethanol for 5 minutes. The slides were washed copiously with running water for 2 minutes and dipped in hematoxylin solution (Thermo Fisher, USA) for 20 seconds. Copious washing was repeated followed by dipping in acid alcohol solution (95% ethanol and 1% hydrochloric acid) five times. The slides were copiously washed again and sequentially dipped in eosin solution (Thermo Fisher, USA) twice before a last round of copious washing. The slides were then immersed sequentially in 70% ethanol solution for 5 minutes, 95% ethanol for 5 minutes, 100% ethanol for 5 minutes, and xylene for 10 minutes. The slides were left to dry on the bench, covered with mounting media (Thermo Fisher, USA) spread evenly over the tissues and protected with coverslips.

Urine collection, monitoring and post-termination analysis

Urine was collected from each mouse under anesthesia before treatment via a 24 G cannula attached to a 1 ml syringe. Routine bodyweight monitoring and observation of all mice were performed to detect mice with 15-20% weight loss and other early termination criteria such as unresponsiveness, unkempt hair coat, hunching, and poor body condition score (**Fig. S15**). Bladders and DLN were harvested, and the organ weights were recorded. Harvested bladders

were stored in -80 °C before RNA extraction for PSA gene detection and quantification using real-time PCR. The presence of tumors in the bladder is defined as PSA gene Ct values < 35. Relative quantification (RQ) are relative fold changes of the PSA gene normalized with the 18sRNA and then compared with a bladder from the control group, which is set as the reference at 1. An initial smaller experiment was performed (n = 5) to detect any nanocarrier or device-related complications and to observe the targeted photodynamic-chemotherapy effects in a low-powered study (**Figure S16**). The cure rate data from the small study (**Table S3**) provided a strong assumption for power analysis of proportions calculations for a larger study to prove treatment efficacy. After the low-powered study, the initial device was updated with an increase in the encapsulation area around the circuitry to safeguard against the perforation damages. The survival and functionality of the improved device were tested in a small batch of tumor implanted mice (**Figure S17**). Once consistent device functionality over a 33-day period was verified, a larger experiment was conducted to prove the efficacies of targeted photodynamic-chemotherapy (50GCG + PDT) with the updated device described in **Figure 2** and targeted chemotherapy-alone (50GCG) compared to saline-treated control (n = 20 per group).

Tumor size calibration curve

MB49 and MB49-PSA cells were mixed at various ratios, with the total cell numbers kept at 2×10^6 (**Figure S14**). RNA was harvested from each sample, and the PSA gene levels were analyzed by qPCR. A tumor size calibration curve was obtained by plotting the delta Ct values, which is the Ct value of the 18RNA gene subtracted from the Ct value of the PSA gene, in GraphPad software. The tumor sizes of each mouse were interpolated from the calibration curve by inputting their delta Ct values in GraphPad and analyzed using nonlinear regression analysis.

Synthesis, characterization, and optimization of glycosylated nanocarriers

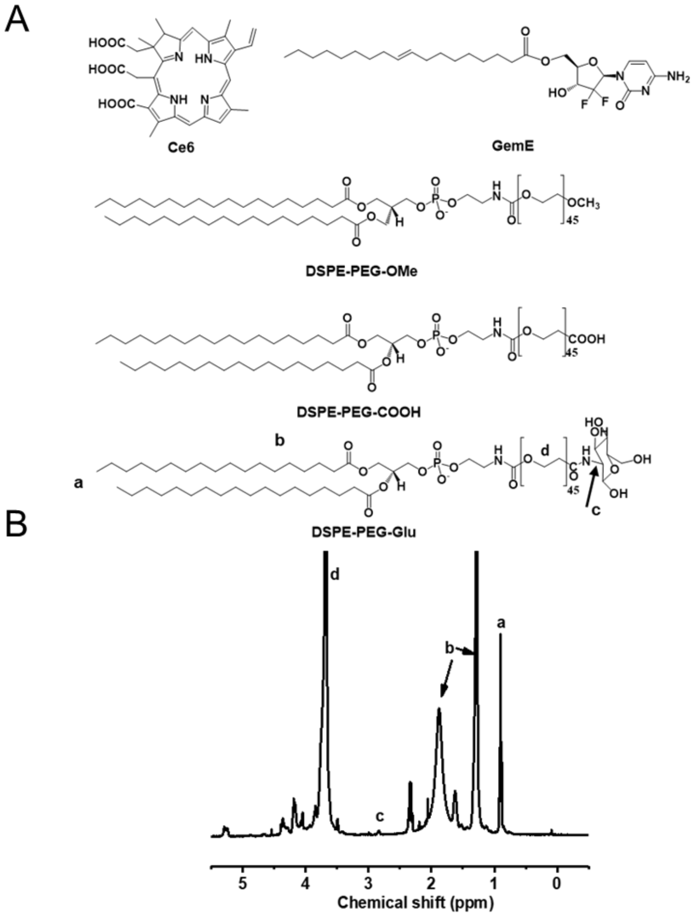

Figure S1.
(*A*) Chemical structure of Ce6, GemE, DSPE-PEG-OMe, DSPE-PEG-COOH, and DSPE-PEG-Glu. (*B*) ^{1}H NMR spectrum (in $CDCl_3$) of DSPE-PEG-Glu, and assignment of H resonance peaks according to DSPE-PEG-Glu structure in (*A*).

DSPE-PEG-Glu was synthesized by conjugating the $-NH_2$ group of glucosamine to the -COOH group of DSPE-PEG-COOH using EDC/NHS. The expected structure of DSPE-PEG-Glu is shown in **Fig. S1*A***. This manner of conjugation is optimal for targeting GLUTs because the bulky substituent at the C-2 position of glucosamine will not interfere significantly with the interactions between glucose and GLUTs [3]. The ^{1}H NMR spectrum of the obtained product

with the various H resonance peak assignments is shown in **Figure S1*B***. In particular, the peaks at 0.9 ppm (t) and 2.84 ppm (m) in the ^{1}H NMR spectrum can be ascribed to the methyl groups of DSPE and the H atom at the C-2 position of glucosamine, respectively, while the peak **d** at 3.67 ppm (b) can be ascribed to the backbone of PEG and other H atoms of glucosamines. The area ratio of the peaks at 0.9 ppm to 2.84 ppm is 6:1, which is consistent with the expected structure of DSPE-PEG-Glu, thus confirming its successful synthesis.

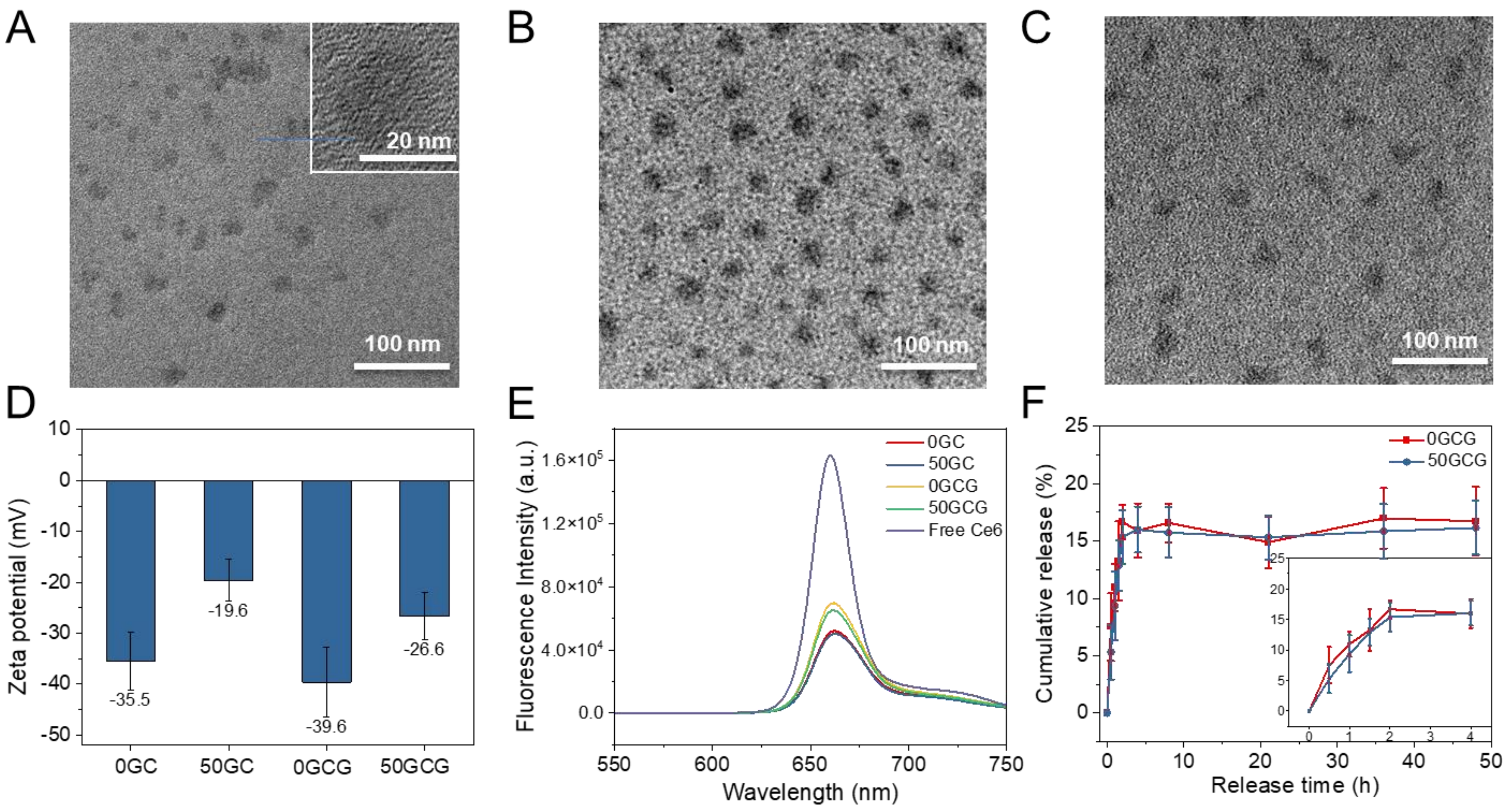

Figure S2.

(*A*) TEM and HRTEM images of Ce6 and GemE co-loaded non-glycosylated nanocarrier, 0GCG. TEM images of 50GCG (*B*) and 0GCG (*C*) stored at 4 over a period of 258 days. (*D*) Zeta potential of 0GC, 50GC, 0GCG, and 50GCG. (*E*) Fluorescence spectra of 0GC, 50GC, 0GCG, 50GCG and free Ce6 (all of 5 μM Ce6 concentration) in PBS with 0.1% DMSO. (*F*) Ce6 release from 0GCG and 50GCG incubated in artificial urine. Data are presented as mean ± SD (n = 3).

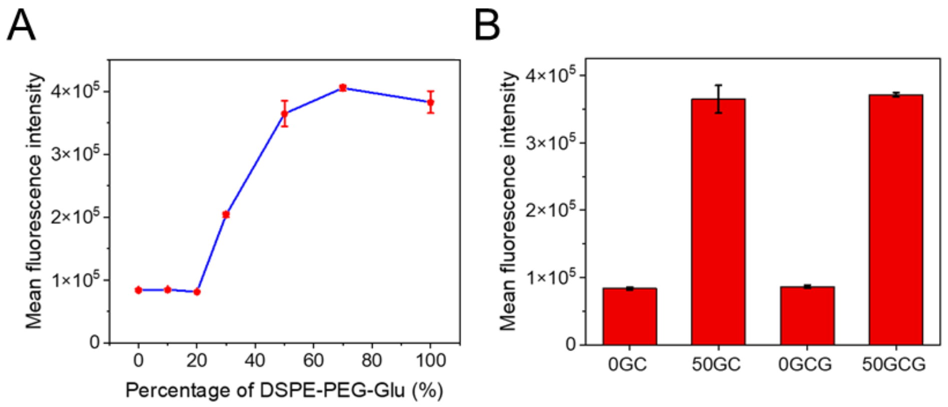

Figure S3.
Flow cytometry of UMUC3 cells after incubation for 2 h with glycosylated carriers. (*A*) Mean fluorescence intensity (Ex = 630 nm, Em = 660 nm) as a function of the percentage of DSPE-PEG-Glu in the Ce6-loaded glycosylated carriers. Data are presented as mean ± SD (n = 3). (*B*) Mean fluorescence intensity (Ex = 630 nm, Em = 660 nm) after incubation with 0GC, 50GC, 0GCG and 50GCG. Data are presented as mean ± SD (n = 3).

For **Figure S3*A***, increasing the DSPE-PEG-Glu content beyond 20% resulted in further Ce6 uptake. It is likely that when the content of DSPE-PEG-Glu is low, the glucose molecules are shielded by the PEG layer on the surface of micelles [4]. Hence, increasing the DSPE-PEG-Glu content exposed the glucose molecules, enabling the targeting of GLUTs on the cell membrane.

Figure S3*B* indicates the co-loading of GemE with Ce6 did not affect the targeting capability of the nanocarrier since UMUC3 cells treated with 0GCG and 50GCG (GemE co-loaded with Ce6) resulted in similar mean fluorescence intensity as the respective carrier loaded with Ce6 only (0GC and 50GC). The mean intracellular Ce6 fluorescence in 50GC is ~4.5-fold higher than 0GC.

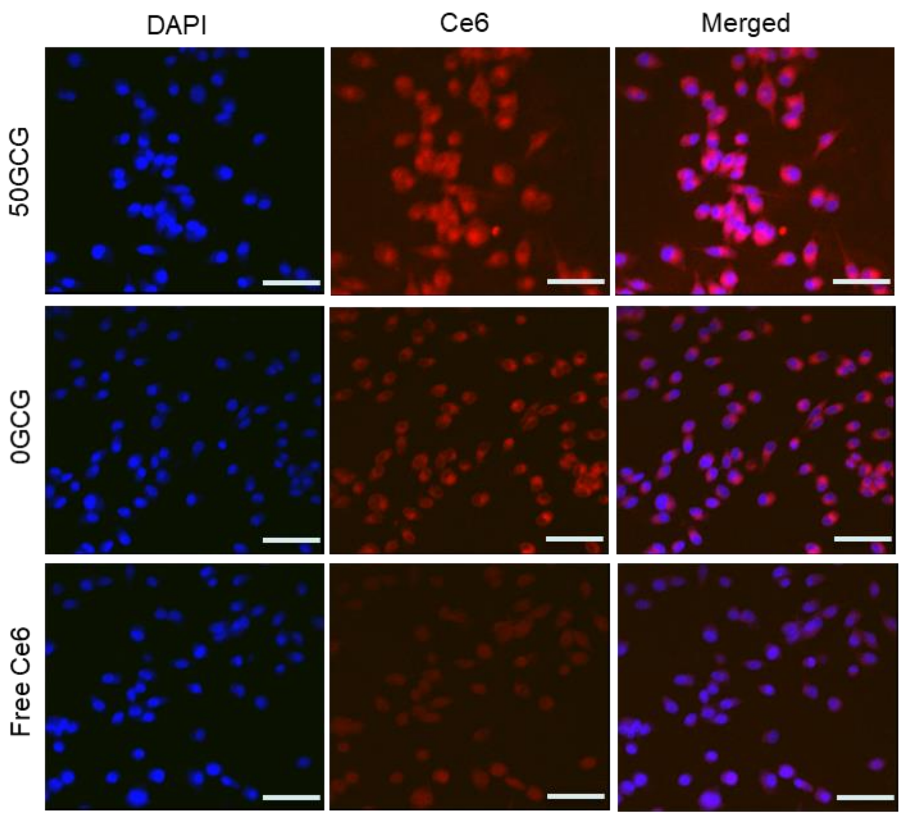

Figure S4.
CLSM images of UMUC3 cells incubated with 0GCG, 50GCG and free Ce6 for 2 h with cell nuclei stained with DAPI. Objective lens, 40×. Scale bar, 100 μm.

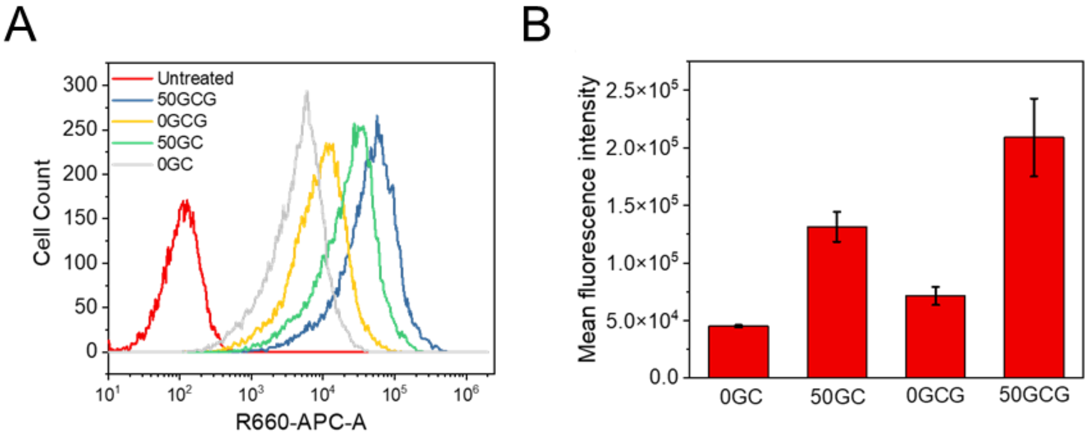

Figure S5.
Flow cytometry of MB49 cells after incubation with DSPE-PEG carriers. (*A*) Flow cytometry of MB49 cells after incubation for 2 h with 0GC, 50GC, 0GCG, and 50GCG. (*B*) Mean

fluorescence intensity of MB49 cells (Ex=630 nm, Em=660 nm) after incubation with 0GC, 50GC, 0GCG, and 50GCG. Data are presented as mean ± SD (n = 3).

Evaluation of targeting efficacy and cytotoxicity

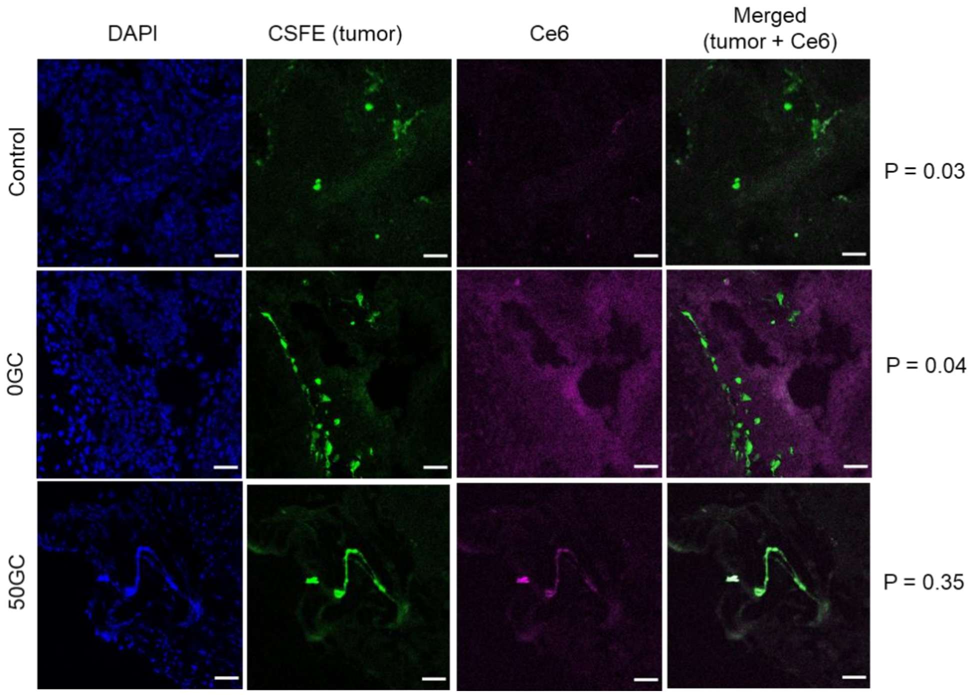

Figure S6.
In vivo binding of Ce6-loaded carriers to implanted bladder tumors. MB49-PSA cells were labeled with 10 µM of CFSE dye (green) and implanted in mouse bladders. Objective lens, 20×. Scale bar, 100 µm.

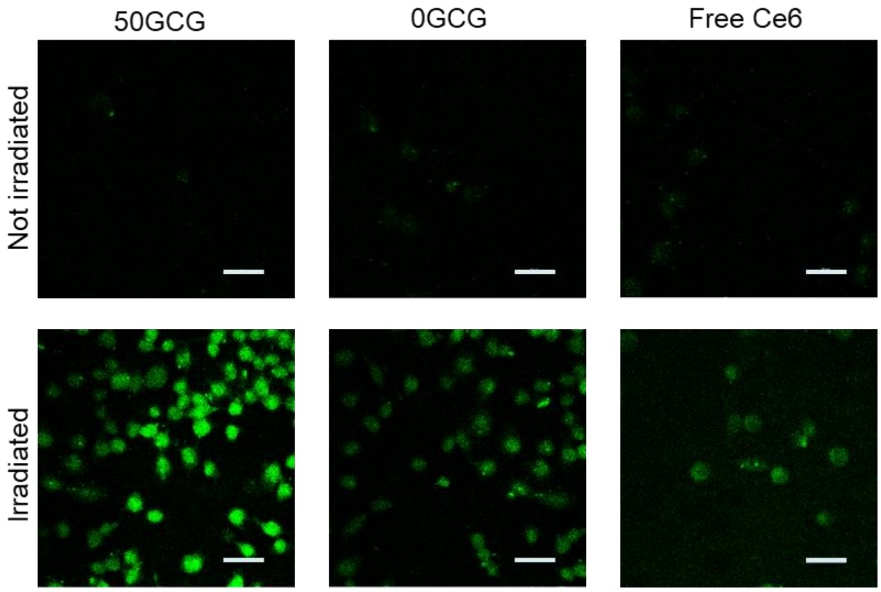

Figure S7.
Fluorescence microscopy images of UMUC3 cells incubated with 50GCG, 0GCG, and free Ce6 for 2 h and then treated with DCFH-DA with and without 660 nm laser irradiation. Objective lens, 20×. Scale bar, 50 μm.

DCFH-DA was applied to detect intracellular ROS generation in the irradiated cells. **Figure S7** shows that after 660 nm laser irradiation, the cells treated with 50GCG possessed the highest intracellular DCF green fluorescence intensity compared with cells treated with 0GCG and free Ce6, demonstrating that the glycosylated carriers increase the uptake of the loaded Ce6 by the UMUC3 cells, resulting in increased intracellular ROS generation.

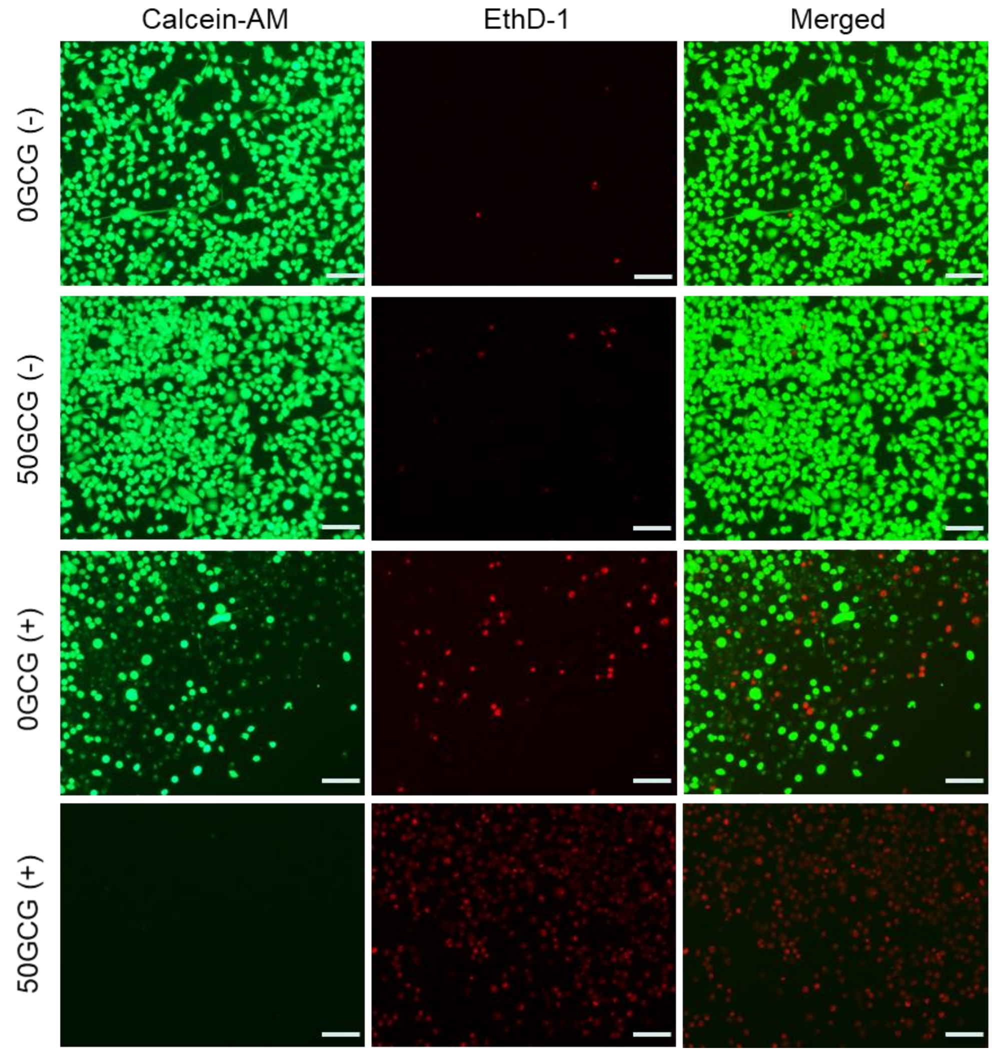

Figure S8.
Live/dead staining results for visualizing the increased cytotoxicity of 50GCG compared with 0GCG. UMUC3 cells were incubated with 0GCG or 50GCG for 2 h without (denoted as 0GCG (-) or 50GCG (-)) and with (denoted as 0GCG (+) or 50GCG (+)) subsequent 660 nm laser irradiation (125 mW cm^{-2}) for 3 min. Calcein-AM was used to stain live cell green, while ethidium homodimer-1 was used as a red dye to visualize dead cells. Objective lens, 10×. Scale bar, 100 µm.

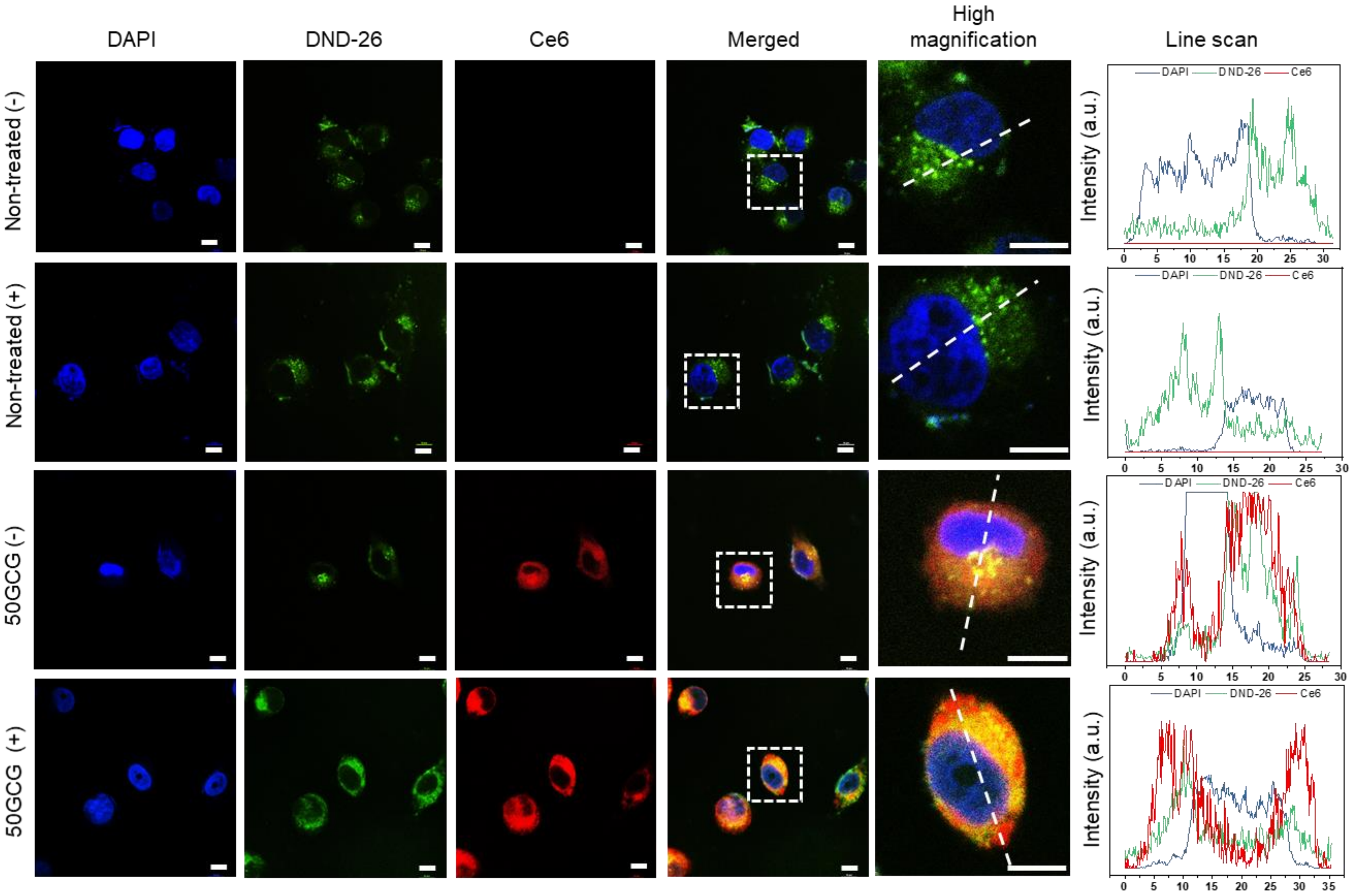
DAPI
DND-26
Ce6
Merged
High magnification
Line scan
Non-treated (-)
Non-treated (+)
50GCG (-)
50GCG (+)
Intensity (a.u.)
DAPI
DND-26
Ce6

Figure S9.
CLSM images of UMUC3 cells incubated for 2 h in the absence of nanocarriers with (denoted as Non treated (+)) and without (denoted as Non treated (-)) 660 nm laser irradiation (250 mW cm^{-2}) for 30 s, UMUC3 incubated with 50GCG for 2 h with (denoted as 50GCG (+)) and without 660 nm (denoted as 50GCG (-)) laser irradiation (250 mW cm^{-2}) for 30 s. Cell nuclei were stained with DAPI and lysosomes were stained with LysoTracker green DND-26. Luminescence intensity scan along the white dashed line marked on the high magnification merged images is shown in the right panel of each figure. Objective lens, 100×. Scale bar, 10 µm.

To better understand the synergistic effect, the fate of 50GCG after endocytosis was studied. Without irradiation, the fluorescence of Ce6 in 50GCG-treated cells overlapped predominantly with the green DND-26-stained endosomes (**Figure S9**). However, after 660 nm laser irradiation (30 s), the green DND-26-stained endosome granules were damaged, and Ce6 fluorescence was detected in the cytoplasm. In contrast, the endosomes of the cells without 50GCG treatment remained intact under laser irradiation, similar to those of non-irradiated cells. These results indicated that the PCI effect enabled the escape of 50GCG micelles from the endosome allowing GemE to exert its chemotherapeutic effects in the cytoplasm and nucleus. GemE release from the micelles would be more challenging without the PCI effect due to their hydrophobic nature. Thus, ROS generated by PDT not only has direct toxic effects on the 50GCG-treated cells and also indirectly promotes chemotherapy by the co-loaded GemE, thereby resulting in the treatment synergy (**Figure 1*C***).

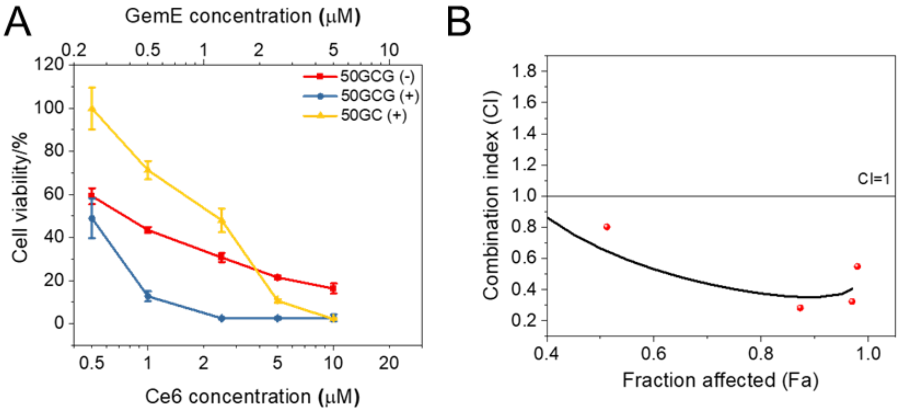

Figure S10.

In vitro cell toxicity of 50GC and 50GCG against MB49 cells and evaluation of the combined effect of the targeted photodynamic-chemotherapy strategy. (*A*) MB49 cell viability after 2 h incubation with 50GCG and 50GC followed by 660 nm laser irradiation (250 mW cm^{-2}) for 5 min (denoted as 50GCG (+) and 50GC (+), respectively), and 50GCG without laser irradiation (denoted as 50GCG (-)). Data are presented as mean ± SD (n = 3). (*B*) CI versus Fa plot of 50GCG with 660 nm laser irradiation against MB49 cells. CI values were calculated using the cell viability results obtained with 50GCG with and without light irradiation and 50GC with light irradiation.

Wireless device fabrication, characterization, and implantation

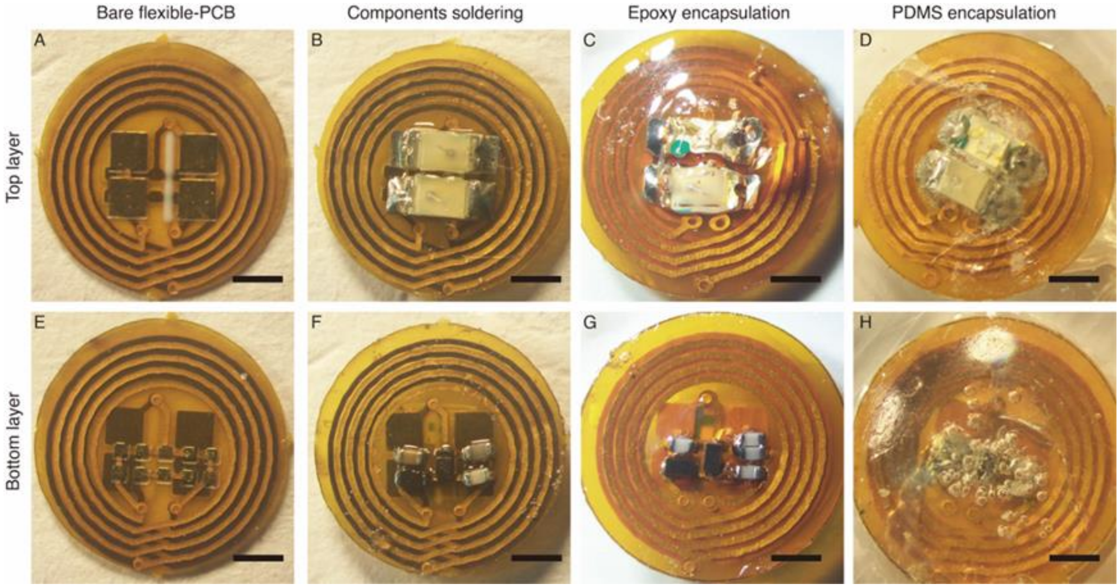

Figure S11.
Wireless photodynamic therapy (PDT) device fabrication process. The spiral coils for wireless powering and footprints for electronic components were designed on a 2-layer flexible printed circuit board (FPCB). Photo (*A*) presents the bottom layer including the two light-emitting devices (LED) for drug activation. (*B*) The rectifier circuit, composed of Schottky diodes and capacitors, was placed in the top layer. (*C*, *D*) The components were soldered in the FPCB by using a micro-soldering system with silver paste. (*E*, *F*) After the soldering, both the top and bottom layers were encapsulated by epoxy to protect the electronic components against moisture and mechanical damage. (*G*, *H*) In the last fabrication step, the device was fully coated with polydimethylsiloxane (PDMS), a widely used biocompatible material. Scale bar 1 mm.

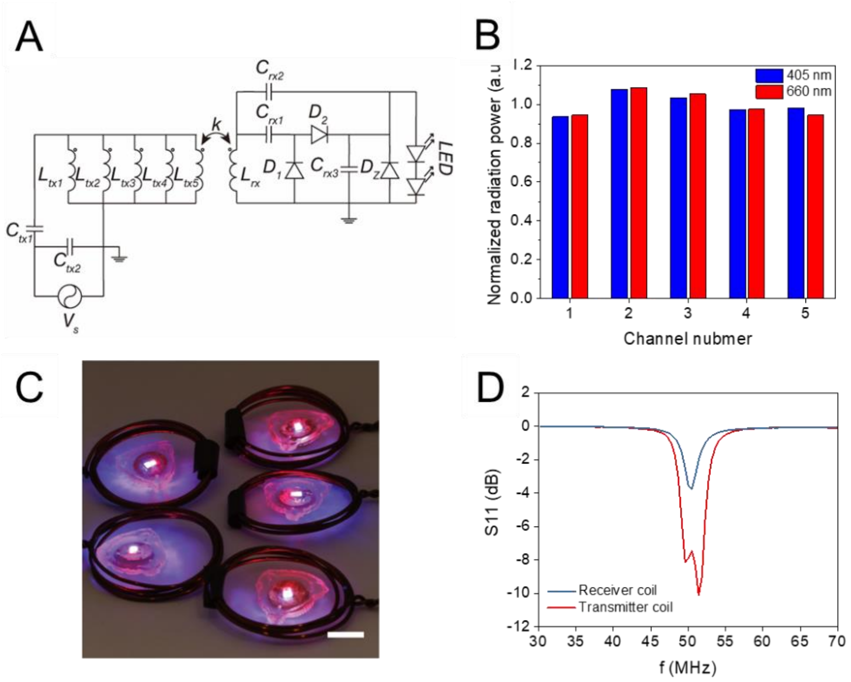

Figure S12.
Transmitter coil array for multiple activations. (*A*) Multiple activated wireless devices using coil array. (*B*) Circuit diagram of a wireless system including transmitter coil array and implantable device. (*C*) Radiant power output of a single wireless device activated using the various channels in the coil array. Scale bar, 5 mm. (*D*) Reflection coefficient of transmitter coil array and receiver coil.

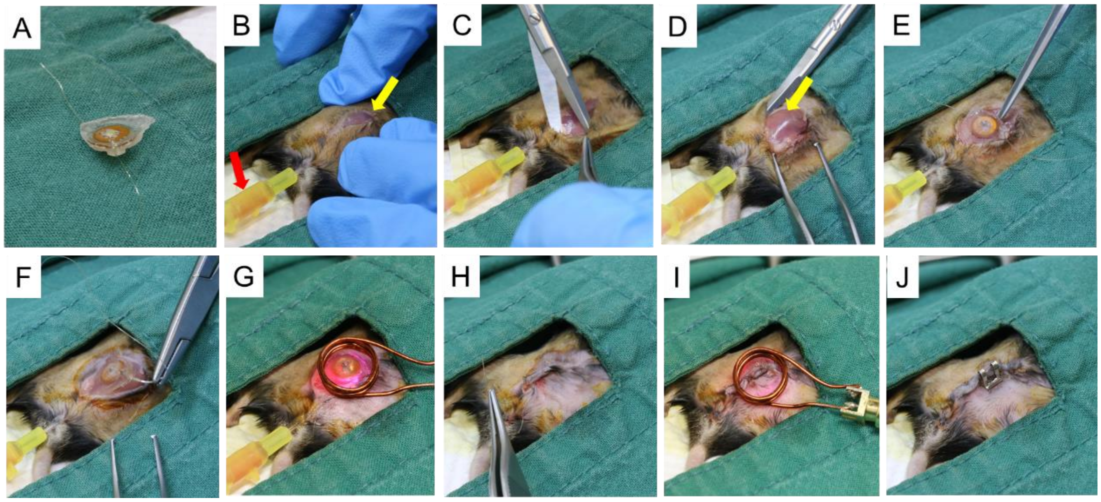

Figure S13.
Implantation of a wireless micro-LED device for mouse orthotopic bladder cancer model. (*A*) The micro-LED device was tethered with suture material on one end of the suture hole. (*B*) The bladder was inflated with 0.1 mL of saline using a 24 G plastic cannula (red arrow). A small (5-8 mm) midline incision was made on the skin (yellow arrow). (*C*) Blunt separation of the skin from the attached muscular tissue was done using a pair of tissue forceps and curved scissors. (*D*) The inflated bladder was visible against the abdominal muscle layer (yellow arrow). (*E*) The device was placed micro-LED side down facing the bladder. (*F*) It was anchored on the abdominal muscle by 2-3 turns of the tethered suture material. The device was anchored using all three suture holes provided on the encapsulation material. (*G*) The device was briefly activated before wound closure. (*H*) The wound was closed with horizontal mattress sutures beginning from the middle of the wound area. The edges were then sealed similarly with the mattress suturing technique. (*I*) The device was activated again after the wound closure was completed. (*J*) The wound closure was reinforced with Vetbond, followed by applying one wound clip to secure the wound apposition.

Device testing and evaluation of treatment efficacy using mouse orthotopic bladder cancer model

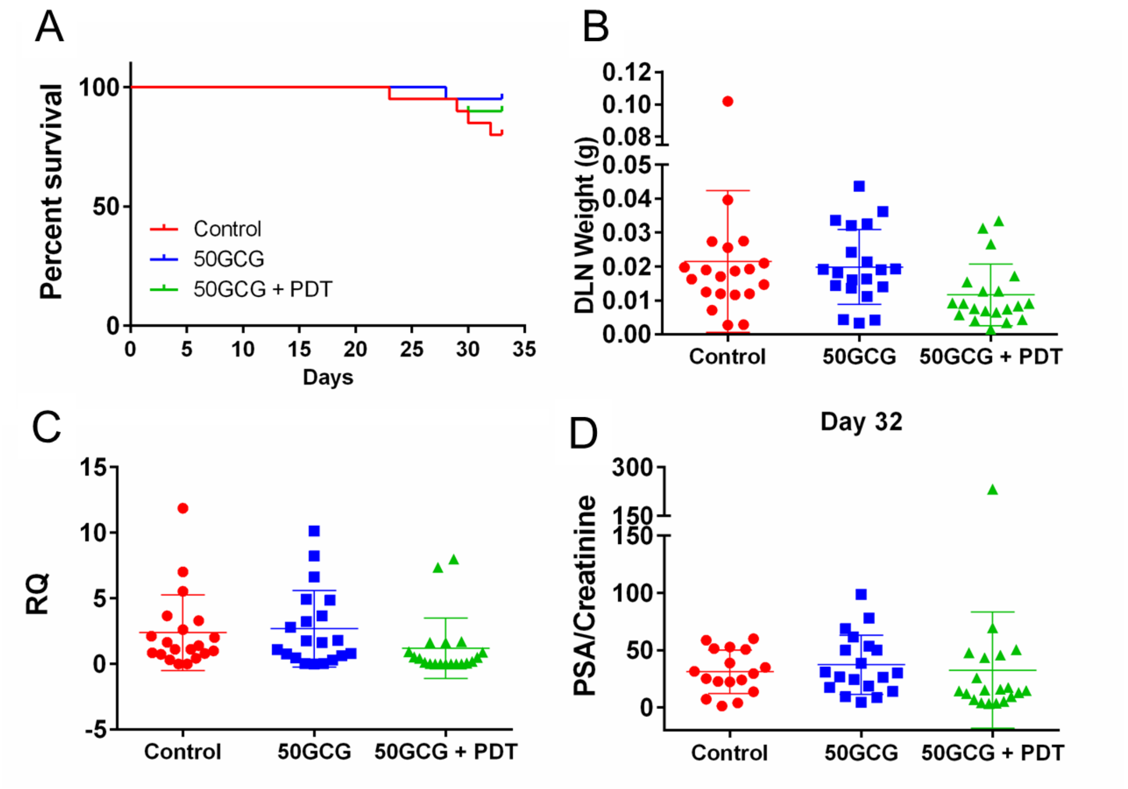

Figure S14.

In vivo efficacy of 50GCG combined with wireless PDT in a mouse orthotopic bladder cancer model. (*A*) Kaplan-Meier analysis of the overall survival of the groups of animals in the *in vivo* efficacy experiment. (*B*) Weight of draining lymph nodes harvested after termination. (*C*) Relative quantification of PSA gene expression in the bladder tissues measured by qPCR. (*D*) Free PSA/creatinine levels in the urine samples obtained on Day 32 of the experiment schedule. Data are presented as mean ± SD (n = 20).

A

MB49-PSA	MB49
0	2000000
100	1999900
1000	1999000
10000	19990000
100000	1900000
1000000	1000000
2000000	0

B

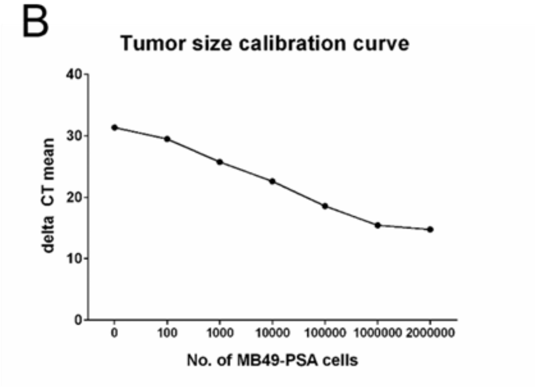

Figure S15.

Tumor size measurement. (*A*) MB49-PSA and MB49 cells were mixed according to the numbers in each row. RNA from the mixed cell population was extracted and used for PSA gene detection using qPCR. (*B*) The delta Ct calibration curve from the mixed cells samples was used to interpolate the delta Ct mean values from each bladder sample in the final efficacy experiment.

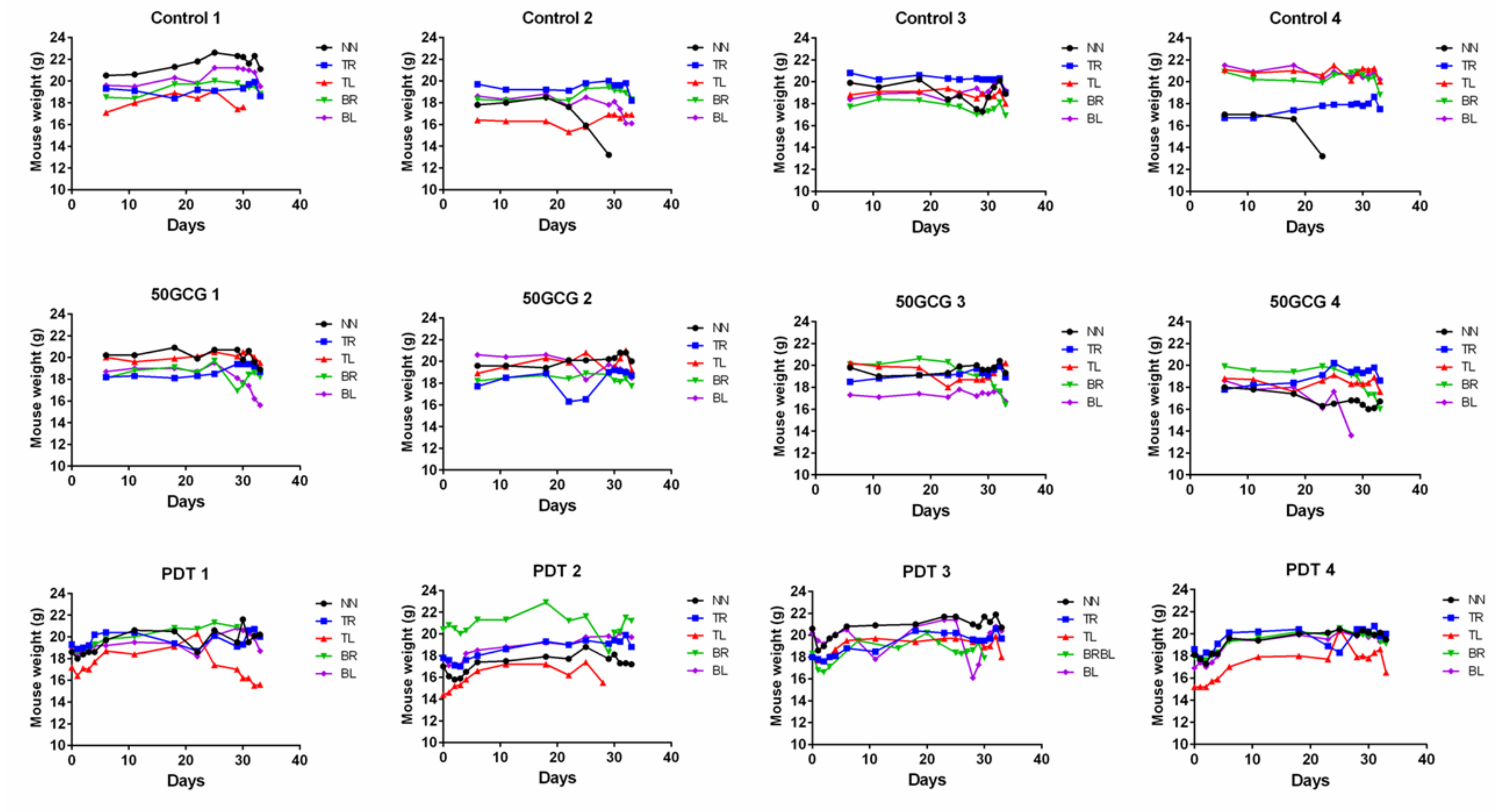

Figure S16.
Bodyweight monitoring of all the mice in the final efficacy experiment. Mice were routinely monitored to observe general well-being and to detect signs of early termination criteria.

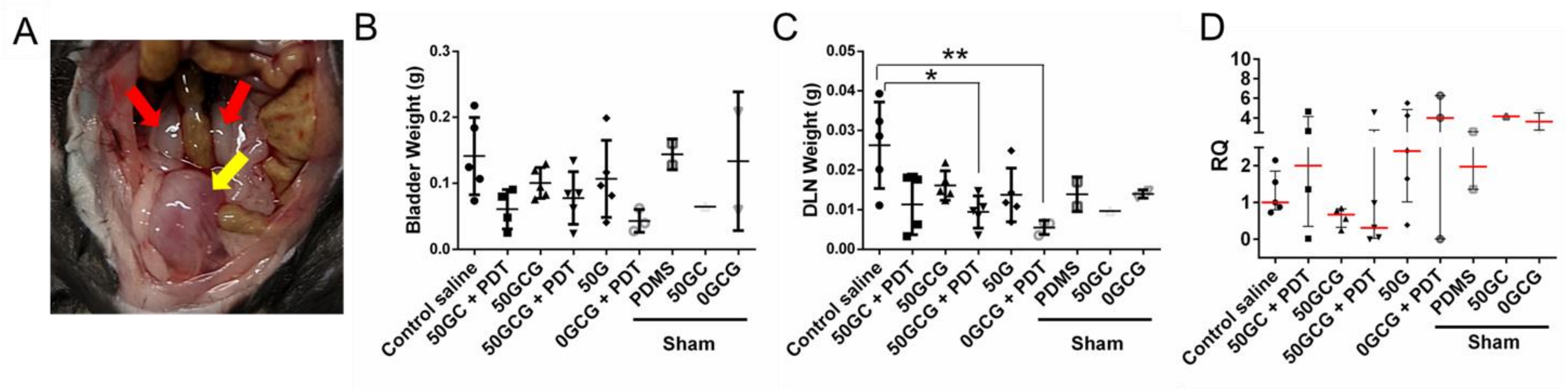

Figure S17.

Initial small-batch efficacy testing of DSPE-PEG-Glu formulations combined with wireless PDT in a mouse orthotopic bladder cancer model. The experiment followed the same schedule as depicted in **Figure 4*A***. Mice were treated with various formulations at 0.2 mg ml^{-1} intrabladder concentrations (empty glycosylated nanocarrier; glycosylated nanocarrier with Ce6, 50GC; glycosylated nanocarrier with GemE and Ce6, 50GCG; non-glycosylated nanocarrier with GemE and Ce6, 0GCG) for 2 h followed by PDT with implanted wireless device (405 nm/660 nm) for 20 min at input power 100 mW. Devices in implanted mice that failed to activate during the first PDT session (3/15, 20%) were regrouped as shams. (*A*) Position of the draining lymph nodes (red arrows) closest to the bladder (yellow arrow)) harvested after termination. (*B*) Harvested bladder and (*C*) Draining lymph nodes weight. Data represented as mean ± SD. (*D*) Relative quantification of PSA gene detected in the bladder with qPCR. Data represented as median ± interquartile range. 8 µM GemE; 16 µM Ce6. The experiment was done once. Data are presented as mean ± SD (n = 5). * $p < 0.05$, ** $p < 0.01$

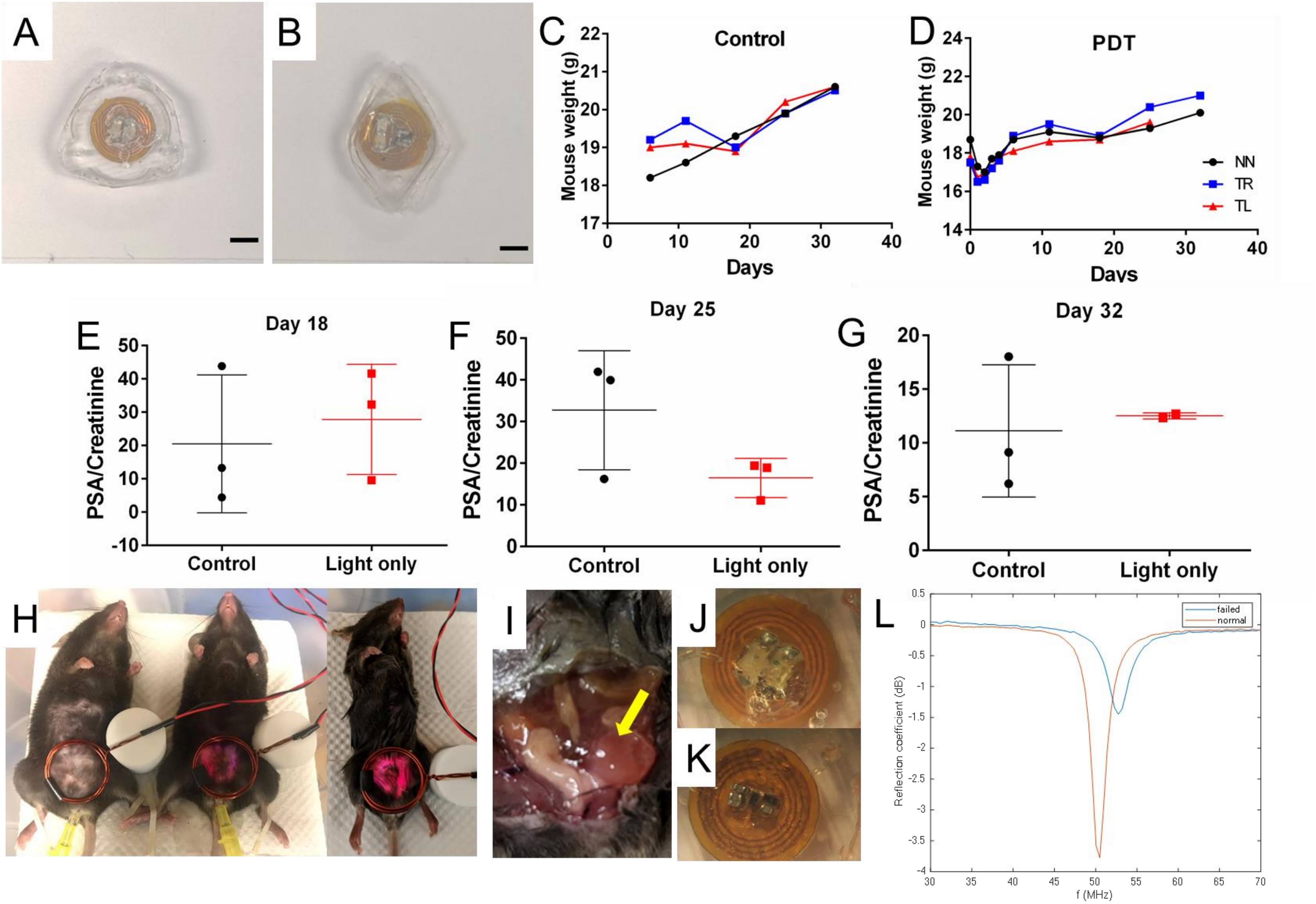
A
B
C
Control
Mouse weight (g)
Days
D
PDT
Mouse weight (g)
Days
NN
TR
TL
E
Day 18
PSA/Creatinine
Control
Light only
F
Day 25
PSA/Creatinine
Control
Light only
G
Day 32
PSA/Creatinine
Control
Light only
H
I
J
K
L
failed
normal
Reflection coefficient (dB)
f (MHz)

Figure S18.
In vivo survival testing of the updated device in mice for 33 days. (*A*) New device version. (*B*) Older device version. Scale bar, 5 mm. Both devices were similarly fabricated on the flexible PCB board, but the new device had an increased area of PDMS encapsulation surrounding the circuitry to increase robustness and protection. Three mice were implanted with the new device. MB49-PSA tumor cells were implanted in the bladders of both control and device implanted mice six days later. The mice underwent a similar treatment schedule as outlined in **Figure 4*A*** with intrabladder saline treatment only. Wireless light delivery was performed in device implanted mice for 30 min per session at input power 121 mW. (*C*) Weight of control and (*D*) Light-only treated mice during the experiment; Free PSA detection in the urine on (*E*) Day 18, (*F*) Day 25 and (*G*) Day 32. Weekly urinary fPSA levels did not indicate regression of tumor growth in the light-only group. (*H*) Activation of the devices during the last light delivery session. The device in one mouse failed during the 4th session. One mouse died two days before the last treatment, but the implanted device was still functioning. (*I*) Post-mortem observation of the mouse that died early revealed a blocked and thickened bladder, indicating possible advanced disease. The outline of the tumor is visible against the full bladder (yellow arrow). (*J*) Image of LED side and (*K*) rectifier side of the failed device. No mechanical damage was found on the surface. (*L*) Reflection coefficients of a functioning (red line) and the failed device (blue line). The shift in reflection coefficient in the failed device could result from the detachment of the soldered components from the PCB board.

Table S1. Design parameters of the wireless powering system shown in **Figure S11*A*.**

Parameters	value	Model	Manufacturer
L_{txn}	512.5 nH	-	-
C_{tx1}	72.7 pF<br>(68 + 4.7)	04025A680FAT2A<br>04025A4R7KAT2A	AVX Corporation
C_{tx2}	420 pF<br>(2x100 +220)	04025A101JAT2A<br>CGA2B2C0G2A221J050BA	AVX Corporation<br>TDK Corporation
L_{rx}	376.5 nH	-	Interhorizon Corporation Pte Ltd
C_{rx1}	100 pF	06031A101JAT2A	AVX Corporation
C_{rx2}	27 pF	GCM0335C1H270JA16D	Murata Electronics
C_{rx3}	220 pF	C0603C221K1RACTU	KEMET
D_n	Schottky diode	BAT2402LSE6327XTSA1CT-ND	Infineon Technologies
LED (660 nm)	Red LED	SML-LX0603SRW-TR	Lumex
LED (405 nm)	Blue LED	SM0603UV-400	Bivar Inc

Table S2. Descriptive statistics of the tumor size data between groups

	($\times 10^4$ cells)		
	Control	50GCG	50GCG + PDT
Minimum	0	0	0
25% Percentile	10.6	3	0
Median	27.15	8.95	3.6
75% Percentile	55.05	31.83	10.88
Maximum	130.3	106.5	93.4
Mean	38.81	22.03	13.63
Std. Deviation	34.36	30.34	27.46
Std. Error of Mean	7.683	6.784	6.14

The tumor size categories were based on the statistics from the control group. Small tumors were defined as $< 20 \times 10^4$ cells (1/2 of mean), and large tumors were defined as $> 20 \times 10^4$ cells.

Table S3. Cure rates of respective treatment groups in initial small batch efficacy test.

Group	Cure Rates (%)
Control	0/5 (0)
50GCG	0/5 (0)
50G	0/5 (0)
Sham (PDMS)	0/2 (0)
Sham (50GC)	0/1 (0)
Sham (0GCG)	0/2 (0)
50GC + PDT	1/4 (25)
50GCG + PDT	2/5 (40)
0GCG + PDT	1/3 (33.3)

SI References

[1] N. Sarigul, F. Korkmaz, İ. Kurultak, *Sci. Rep.* **2019**, *9* (1), 1.
[2] a) T.-C. Chou, *Pharmacol. Rev.* **2006**, *58* (3), 621; b) T.-C. Chou, P. Talalay, *Advances in enzyme regulation* **1984**, *22*, 27; c) T.-C. Chou, R. J. Motzer, Y. Tong, G. J. Bosl, *J. Natl. Cancer Inst. Monogr.* **1994**, *86* (20), 1517.
[3] a) E. C. Calvaresi, P. J. Hergenrother, *Chem. Sci.* **2013**, *4* (6), 2319; b) M. Patra, S. G. Awuah, S. J. Lippard, *Journal of the American Chemical Society* **2016**, *138* (38), 12541.
[4] H. Zhou, Z. Fan, P. Y. Li, J. Deng, D. C. Arhontoulis, C. Y. Li, W. B. Bowne, H. Cheng, *ACS nano* **2018**, *12* (10), 10130, https://doi.org/10.1021/acsnano.8b04947.
